# Smart‐Responsive Multifunctional Therapeutic System for Improved Regenerative Microenvironment and Accelerated Bone Regeneration via Mild Photothermal Therapy

**DOI:** 10.1002/advs.202304641

**Published:** 2023-11-07

**Authors:** Minhao Wu, Huifan Liu, Dan Li, Yufan Zhu, Ping Wu, Zhe Chen, Feixiang Chen, Yun Chen, Zhouming Deng, Lin Cai

**Affiliations:** ^1^ Department of Spine Surgery and Musculoskeletal Tumor Zhongnan Hospital of Wuhan University 168 Donghu Street, Wuchang District Wuhan Hubei 430071 P. R. China; ^2^ Department of Anesthesiology Research Centre of Anesthesiology and Critical Care Medicine Zhongnan Hospital of Wuhan University Wuhan Hubei P. R. China; ^3^ Department of Neonatology, Xianning Central hospital School of Basic Medical Sciences, Xianning Medical College, Hubei University of Science and Technology Xianning Hubei 437100 P. R. China; ^4^ Research Units of Clinical Translation of Cell Growth Factors and Diseases Research Chinese Academy of Medical Science Zhejiang 325000 P. R. China; ^5^ Department of Biomedical Engineering and Hubei Province Key Laboratory of Allergy and Immune Related Disease TaiKang Medical School (School of Basic Medicine Sciences) Wuhan University Wuhan 430071 P. R. China

**Keywords:** angiogenesis, bone regeneration, immune microenvironment, mild photothermal activity, on‐demand delivery, smart release hydrogel

## Abstract

The treatment of bone defects remains a substantial clinical challenge due to the lack of spatiotemporal management of the immune microenvironment, revascularization, and osteogenic differentiation. Herein, deferoxamine (DFO)‐loaded black phosphorus nanosheets decorated by polydopamine layer are prepared (BPPD) and compounded into gelatin methacrylate/sodium alginate methacrylate (GA) hybrid hydrogel as a smart‐responsive therapeutic system (GA/BPPD) for accelerated bone regeneration. The BPPD nanocomposites served as bioactive components and near‐infrared (NIR) photothermal agents, which conferred the hydrogel with excellent NIR/pH dual‐responsive properties, realizing the stimuli‐responsive release of DFO and PO_4_^3 −^ during bone regeneration. Under the action of NIR‐triggered mild photothermal therapy, the GA/BPPD hydrogel exhibited a positive effect on promoting osteogenesis and angiogenesis, eliminating excessive reactive oxygen species, and inducing macrophage polarization to the M2 phenotype. More significantly, through macrophage M2 polarization‐induced osteoimmune microenvironment, this hydrogel platform could also drive functional cytokine secretion for enhanced angiogenesis and osteogenesis. In vivo experiments further demonstrated that the GA/BPPD system could facilitate bone healing by attenuating the local inflammatory response, increasing the secretion of pro‐healing factors, stimulating endogenous cell recruitment, and accelerating revascularization. Collectively, the proposed intelligent photothermal hydrogel platform provides a promising strategy to reshape the damaged tissue microenvironment for augmented bone regeneration.

## Graphical abstract

A smart‐responsive multifunctional therapeutic system composed of DFO‐loaded BP nanosheets decorated by PDA layer and GA composite hydrogel is designed and constructed to achieve the stimuli‐responsive release of DFO and PO_4_^3−^ under the action of NIR‐triggered mild photothermal treatment for spatiotemporal management of the immune regulation, revascularization, and osteogenic differentiation during bone healing.

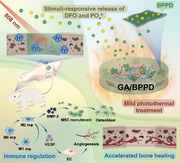

## 1 Introduction

With the increase in the aging population, the incidence of bone fractures and orthopedic‐related injuries is rising every year, resulting in enormous medical and economic burdens as well as impaired quality of life.^[^ ^1^ ^]^ Generally, the regeneration of damaged bone tissue, especially for large bone defects, is a complex cascade reaction process, requiring initiation of the immune system and microenvironment regulation, activation of vascular ingrowth, and subsequent osteogenic differentiation. At the initial stage of bone defect repair, the implantation of bone graft substitute materials triggers local inflammation and the immune microenvironment, recruiting multiple immune cells (e.g., neutrophils, natural killer cells, macrophages, dendritic cells, and T and B lymphocytes) to participate in inflammation development and regulation.^[^ ^2^ ^]^ Among these, macrophages, as the most common effector immune cells at the inflammatory phase, participate in all phases of bone healing. According to classical theory, macrophages can be divided into proinflammatory macrophages (M1) and anti‐inflammatory macrophages (M2) in response to diverse signal stimuli. More specifically, M1‐type macrophages induce excessive fibrosis and healing disorders by secreting proinflammatory cytokines and reactive oxygen species (ROS), while M2 macrophages tend to promote angiogenesis and osteogenic differentiation through the secretion of multiple anti‐inflammatory and pro‐healing cytokines.^[^ ^3^, ^4^ ^]^ Unfortunately, excessive or prolonged inflammation can result in macrophage polarization to a proinflammatory M1 phenotype, with the formation of granulomas and fibrotic scar tissue, which further aggravate the inflammatory response following injury and thus compromise cell proliferation and tissue regeneration. Therefore, it is likely that timely shifting of the macrophage phenotype toward the M2 phenotype (i.e., immunomodulation) at the early stages of bone regeneration would create a suitable immune microenvironment (anti‐inflammatory and M2‐polarizing) for high‐performance bone repair.

Once the proper timing activation of M2 phenotype macrophages is achieved, immediately following vascular reconstruction and osteogenesis are coupled in bone formation.^[^ ^5^ ^]^ Advances in materiobiology have recently suggested that angiogenesis and osteogenesis are two critical events in bone fracture healing and remodeling, in which the development of internal blood vessels is essential for the success of tissue engineering and bone regeneration.^[^ ^6^ ^]^ Furthermore, accelerated vascular ingrowth can provide nutrients and transport metabolites essential for osteogenesis and may recruit osteoprogenitors to the injury area, promoting bone tissue regeneration by establishing cell and nutrient transport channels. Conversely, the impaired blood supply of implanted bone repair material results in delayed tissue healing, which has become a serious obstacle to its application.^[^ ^7^ ^]^ Recent research has also highlighted the importance of neovascularization in skeletal development and bone repair.^[^ ^8^ ^]^ Therefore, a promising strategy for bone augmentation is the utilization of tissue‐engineered biomaterials to recreate a conducive immune microenvironment at the inflammation stage and subsequently coordinate angiogenesis and osteogenesis in an intelligent manner, thereby achieving optimized tissue healing outcomes.

Among various bone tissue engineering scaffolds developed thus far, biocompatible 3D hydrogels have been considered to be excellent artificial bone graft materials owing to their similarity to the extracellular matrix (ECM) in physical and chemical properties as well as their extraordinary capacity to serve as drug loading (encapsulated or bonded) and delivery vehicles.^[^ ^9^ ^]^ The vast majority of previously developed hydrogels have solely been used as carriers to encapsulate various biological products, failing to satisfy the proper timing manipulation of macrophage activation, angiogenesis, and osteogenesis.^[^ ^10^ ^]^ Thus, it is still challenging to develop a biocompatible hydrogel with programmed release‐specific actives to sequentially modulate specific biological functions (e.g., immune response, vessel formation, and subsequent osteogenic differentiation) during bone repair. Although loading exogenous biological agents is a facile and promising strategy that has resulted in significant improvement in the overall performance of biomaterials, improper release behavior leads to delayed or dysfunctional bone regeneration.^[^ ^11^ ^]^ In addition, these therapeutic strategies are limited by initial burst release and easy inactivation of cytokines, drug‐associated complications, high production cost and sophisticated manufacturing. Of note, the dynamic and complex microenvironment in vivo together with the biosafety and short half‐life of these traditional biologically active molecules also make it difficult to match the physiological process of bone repair, inevitably delaying bone healing.^[^ ^12^ ^]^ It is thus of great significance to develop a smart drug delivery system with controlled release for the sequential promotion of immune regulation, vascularization, and recruitment of bone marrow stem cells (BMSCs) and skeletal progenitors, which could meet the complex and ordered process in bone regeneration.

Herein, based on the above introduction, we rationally designed smart‐responsive multifunctional hydrogels with mild photothermal activity to promote bone regeneration through sequential activation of M2 macrophage polarization, angiogenesis, and osteogenesis. The hydrogel platform (denoted as GA/BPPD) was fabricated based on gelatin methacrylate (GelMA)/sodium methacrylic acid alginate (Alg‐MA) hybrid hydrogel (GA) system and further incorporated with black phosphorus (BP)‐based nanocomposite (BPPD), coated with polydopamine (PDA) and loaded with deferoxamine (DFO). As a rising star on the horizon of 2D layered biomaterials, BP nanosheets possess fantastic physiochemical properties, biocompatibility, and superior efficiency in near‐infrared (NIR)‐thermal conversion, leading to their wide application in the fields of biomedical engineering, such as drug delivery, cancer therapy and tissue engineering.^[^ ^13^ ^]^ Additionally, the large specific surface area and unique layered structure endow BP nanosheets with high performance in guest molecule (drug, protein, and gene) delivery, which potentiates the efficient treatment of bone defect‐associated diseases.^[^ ^14^ ^]^ In the physiological environment, the oxidization degradation products of BP nanosheets are usually non‐toxic phosphate ions, which have been reported to promote bone regeneration spontaneously by accelerating cell proliferation, differentiation, and signal transduction, as well as participating in bone metabolism.^[^ ^15^, ^16^ ^]^ Due to various biological functions and easy availability, 2D BP and 2D BP‐based materials combined with a mild NIR‐based photothermal strategy can produce a synergistic effect on promoting bone regeneration, although the efficiency still needs improvement. As reported, BP nanosheets are not stable in aqueous environments and can rapidly degrade into orthophosphate ions under ambient conditions, severely impeding their potential applications in biomedicine.^[^ ^17^ ^]^ Emerging evidence has revealed that the surface modification of BP nanosheets with PDA can not only effectively protect BP from fast degradation and allow for sustained PO_4_^3−^ release that promotes bone regeneration, but also enhance interfacial bonding between pristine BP nanosheets and the polymer matrix to improve stability.^[^ ^18^, ^19^ ^]^ As a synthetic melanin‐like biopolymer, the abundant presence of catechols, amine, and aromatic rings in the PDA structure would enable a high loading and controlled release of therapeutic molecules via hydrogen bonding or π−π stacking interactions.^[^ ^20^ ^]^ In the present work, DFO, as a Food and Drug Administration (FDA)‐approved iron‐chelating agent, was chosen as a bioactive small molecule to be loaded in the PDA‐modified BP (BP@PDA) carrier because of its promoting effects on vascularization and osteogenesis. A growing number of studies have demonstrated that DFO can regulate hypoxia‐inducible factor (HIF‐1α)/vascular endothelial growth cell (VEGF) signaling, thereby enhancing angiogenesis and tissue perfusion.^[^ ^21^ ^]^ More interestingly, each raw material, including BP, PDA, and DFO, was demonstrated to have a direct effect on scavenging free radicals, which could synergistically help protect tissue from oxidative damage and thus suppress reactive oxygen species (ROS)‐induced inflammatory status.^[^ ^22^, ^23^ ^]^ It was recently reported that a pro‐healing immune microenvironment could be achieved by PDA and mild heat stimulation‐mediated macrophage reprogramming.^[^ ^12^ ^]^ We envision that the as‐prepared BPPD nanocomposite with photothermal and therapeutic effects could serve as a smart delivery system to release DFO and PO_4_^3−^ in a pH‐ and thermal‐stimuli‐dependent manner, which holds significant incentives for spatiotemporal manipulation of the immune response, vascular development, and bone regeneration.

**Scheme 1:**
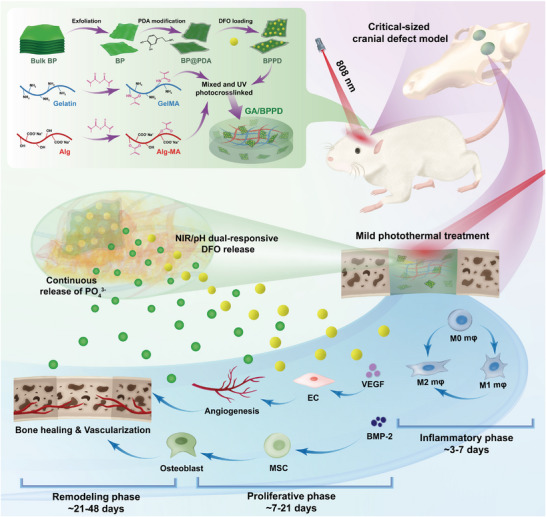
Schematic illustration for fabrication and application of smart‐responsive multifunctional therapeutic system with mild photothermal activity for augmented bone regeneration through spatiotemporal manipulation of the immune microenvironment, stem cell recruitment and vascular development, and osteogenic differentiation throughout the whole healing process.

As shown in **Scheme** **1**, gelatin methacrylate (GelMA)/sodium methacrylic acid alginate (Alg‐MA) hybrid hydrogel (GA) system was selected as the model substrate for cranial defect repair, which could be utilized to reinforce the impaired bone tissue and serve as a carrier for delivering therapeutic drugs/ions from bioactive BPPD nanocomposites. Previous studies have shown that GA hydrogels can not only maintain good stability under physiological conditions but also form a natural 3D polymeric structure in the presence of photoinitiators and ultraviolet light.^[^ ^24^ ^]^ After loading in GA, self‐degradation of the BPPD nanocomposite could be greatly inhibited due to the protective effect of the hydrogel matrix, thereby better exerting its biological activities to meet the sequential demands of cellular response and tissue regeneration. Under internal stimulation of pH and external NIR laser irradiation, sequential release of DFO and PO_4_^3−^ from the BPPD‐loaded GA hybrid hydrogel was observed because these factors significantly affected the desorption of DFO as well as the release of PO_4_^3−^ from BPPD, thereby providing a pro‐regenerative microenvironment. In the present work, we systematically investigated the physicochemical structure and properties, in vitro drug loading and on‐demand release behavior, and in vitro cellular response, including adhesion, proliferation, migration, osteogenic differentiation, angiogenic effects, and macrophage polarization. The subcutaneous implantation of this kind of photothermal hydrogel platform in rats further confirmed its enormous potential in regulating the immune response and promoting revascularization in tissue engineering applications. Finally, the bone regeneration ability of the bioactive nanocomposite hydrogel on bone healing was validated using a critical‐sized calvaria bone defect model under NIR light irradiation. Together, our strategy of integrating bioactive BPPD nanomaterials within a biocompatible GA hydrogel provides a new insight into the design of smart biomaterials for the whole management of bone tissue regeneration (Scheme 1). To the best of our knowledge, this study is the first report that utilizes a smart‐responsive photothermal hydrogel platform to sequentially modulate the immune response, vascularization, and osteogenesis through a combination of physical (mild photothermal treatment) and chemical (drug/ion delivery) interventions.

## 2 Results and Discussion

### 2.1 Preparation and Characterization of the Hybrid Hydrogels

To realize the spatiotemporal regulation of the immune microenvironment, angiogenesis, and osteogenesis, we designed and constructed a bioactive BPPD nanocomposite‐incorporated hybrid hydrogel with strong photothermal effect and desirable NIR‐triggered drug‐releasing capability in the current study. As shown in Scheme 1, the obtained smart delivery hydrogel system can synchronously realize multifunctionality for the treatment of bone defects by combining long‐term physical (photothermal) and continuous chemical (drug/ion delivery) intervention, so as to provide a bone‐friendly microenvironment for sequential activation of M2 macrophage polarization, vascularization, and MSC recruitment, resulting in enhanced bone regeneration.

**Figure 1:**
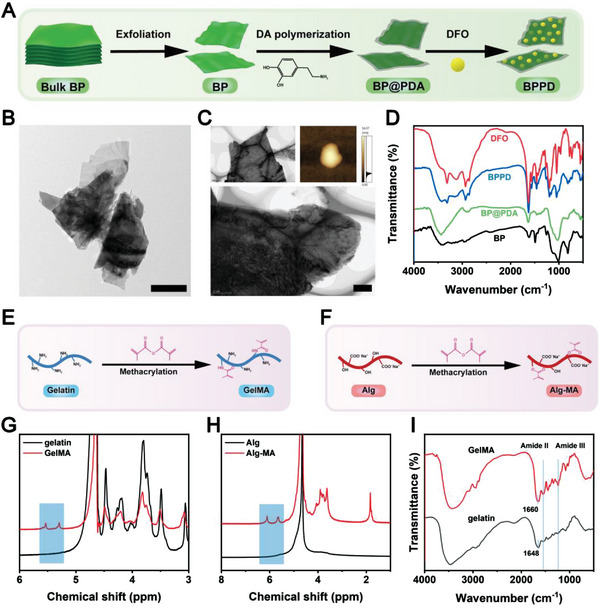
A) Schematic diagram of the fabrication of BPPD. B) TEM and C) AFM images of BPPD. D) FTIR spectra of BP, BP@PDA, BPPD, and DFO. Schematic illustration for the fabrication of E) GelMA and F) Alg‐MA. ^1^H NMR spectra of G) GelMA and H) Alg‐MA. I) FTIR spectra of gelatin and GelMA. Scale bar: 500 nm (B), 1 µm (C).

For preparation of smart‐responsive multifunctional hydrogels, BPPD nanocomposites, which served as NIR/pH dual‐triggered drug release nanoplatforms, were prepared through BP crystal exfoliation, PDA modification, and DFO loading. A schematic illustration of the preparation process of BPPD is shown in **Figure** **1**A. The raw BP nanosheets were first exfoliated by a sonication‐assisted top‐down strategy. PDA was then decorated on the surface of the BP nanosheets by the self‐polymerization reaction of DA using BP nanosheets as a template. Lines of evidence have shown that mussel‐inspired polymerization of DA to form PDA coatings on various biomaterials has emerged as a diverse surface functionalization method for biomedical applications.^[^ ^25^ ^]^ Introducing PDA layer on BP nanosheets could avoid agglomeration induced by high surface energy, achieving not only better physiological stability and interface compatibility with the hydrogel matrix but also good biocompatibility and ROS scavenging capacity as well as strong drug loading capacity.^[^ ^18^, ^26^ ^]^ Previous studies have also shown that the introduction of polyphenols, such as PDA, not only promotes cell migration, adhesion, proliferation, and differentiation but also regulates the inflammatory and immune microenvironment in the early stages of implantation, thus accelerating the subsequent tissue regeneration process.^[^ ^27^, ^28^ ^]^ In the present work, the introduction of PDA endowed this hybrid nanocomposite with improved biocompatibility and simultaneously provided a stable glue to conjugate BP nanosheets and DFO molecules.

Thereafter, the angiogenic drug DFO was introduced to the BP@PDA nanosystem (BPPD) by the interaction between DFO (‐NH_2_) and the rich functional moieties of PDA (e.g., catechols, amine, and aromatic rings). The interaction mechanism between DFO and BP@PDA was mainly attributed to a series of noncovalent forces (physical adsorption, hydrogen bonding, π−π stacking, electrostatic interactions, etc.). It should also be noted that, as a common melanin‐like biopolymer, PDA with enriched catechol groups has excellent photothermal conversion efficiency, which endows BPPD with NIR‐controlled drug release behavior for efficient bone regeneration.

As revealed by transmission electron microscopy (TEM) images, BPPD is a typical 2D multilayered structure with well‐defined edges (Figure 1B). Similarly, the atomic force microscopy (AFM) results reveal the thicknesses of BPPD with a layer height of ≈16 nm (Figure 1C). The successful synthesis of the BPPD nanomaterial was verified by zeta potential and Fourier transform infrared spectrometer (FTIR) analysis. As shown in Figure S1 (Supporting Information), it could be observed that the surface zeta potential of raw BP nanosheets (−30.6 mV) decreased to −56 mV after the in situ polymerization of PDA on BP nanosheets, which was ascribed to the introduction of negatively charged hydroxyl groups from the PDA structure, consistent with previous reports.^[^ ^19^ ^]^ After loading DFO, BP@PDA presented a less negative potential (−42 mV) than unmodified BP@PDA (−56 mV). The increase in zeta potential was ascribed to the immobilization of positively charged DFO onto negatively charged BP@PDA nanosheets. This phenomenon suggested that DFO can be assembled on the surface of BP@PDA nanospheres by electrostatic adsorption, which agrees with previous studies.^[^ ^21^ ^]^ The FTIR spectra of pristine BP exhibited three absorption peaks located at ≈1491, 1010, and 815 cm^−1^, which could be attributed to the stretching vibration of P = O and P‐O bonds, further confirming the successful preparation of BP nanosheets (Figure 1D). After PDA modification, new additional peaks appeared at 1633, 1457 and 1372 cm^−1^ corresponding to the C = C stretching vibration, N‐H scissoring vibration and C‐O stretching vibration of PDA, respectively, demonstrating the successful polymerization of DA in the system. Meanwhile, compared to the FTIR spectrum of BP@PDA, the peaks of BPPD at 1195, 1264, 1461, 1566, and 1628 cm^−1^ are slightly stronger because DFO contains more amide bonds, verifying the successful preparation of the BPPD nanocomposite (Figure 1D).

**Figure 2:**
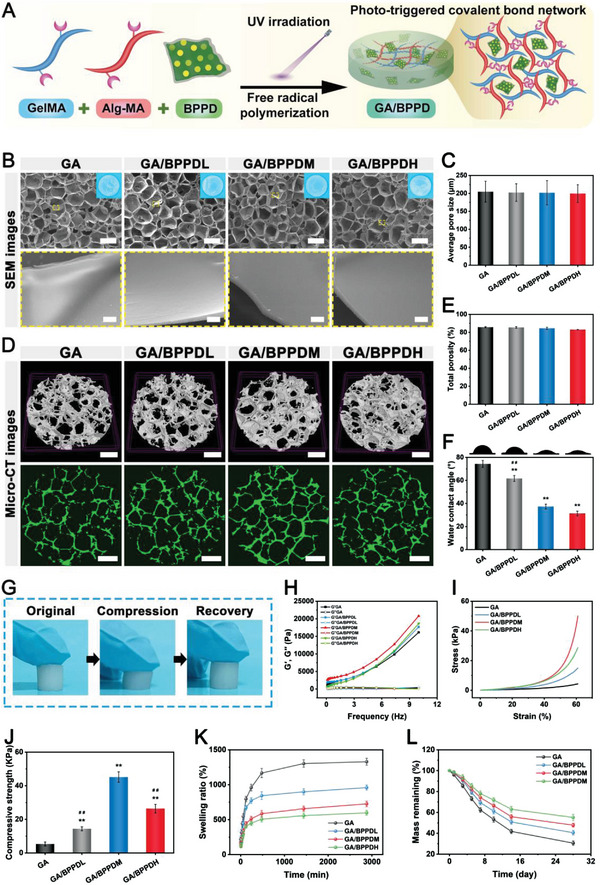
A) Schematic illustration of the fabrication of the GA/BPPD hydrogel. B) SEM and D) micro‐CT images of the lyophilized GA/BPPD hydrogel. Mean C) pore size, E) porosity, and F) water contact angle of the GA/BPPD hydrogel. G) Photographs of the GA/BPPD hydrogel before and after compression. H) Rheological analysis of the GA/BPPD hydrogel. I) Strain–stress curves and J) compressive strength of the GA/BPPD hydrogel. K) Swelling ratio of the hydrogels in PBS solution. L) Degradation behavior of the hydrogels in the presence of 100 mg mL^−1^ lysozyme. Scale bar: 200 µm (low‐magnification SEM images in B), 5 µm (high‐magnification SEM images in B), 300 µm (D). Data are presented as the mean ± SD (*n* = 3). *p < 0.05 and **p < 0.01 indicate significant differences compared with the GA group. ^#^ *p* < 0.05 and ^# #^ *p* < 0.01 indicate significant differences compared with the GA/BPPDM group.

To realize the controlled release of dual factors to match the immune response, angiogenic and osteogenic progressions, different amounts of BPPD nanocomposite were added into the GA pre‐gel solution followed by photo‐polymerization under UV irradiation, forming a photo‐triggered covalent bond network. Both GelMA and Alg‐MA are well‐known photosensitive hydrogels with the advantages of good biocompatibility and biodegradability, which have the ability to mimic the natural ECM of bone to encapsulate bioactive molecules or cells and integrate well with the surrounding tissue.^[^ ^29^ ^]^ The favorable 3D microenvironment provided by the GA hydrogel could accelerate the formation of bone tissue owing to the presence of cell attachment‐promoting arginine‐glycine‐aspartic acid (RGD) sequences.^[^ ^30^ ^]^ As major building blocks for the GA/BPPD hydrogel, photo‐cross‐linkable GelMA and Alg‐MA prepolymers were designed and synthesized by modifying gelatin and Alg with reactive methacrylate groups, as illustrated in Figure 1E,F. The successful preparation of methacrylated gelatin and Alg products was confirmed by proton‐1 nuclear magnetic resonance (^1^H NMR) and FTIR. As shown in Figure 1G,H, the methyl (‐CH = CH_2_) proton peak was observed in the spectra of GelMA (5.46 and 5.67 ppm) and Alg‐MA (5.65 and 6.08 ppm), indicating the successful introduction of double bonds into gelatin and Alg and the successful synthesis of GelMA and Alg‐MA. FTIR spectra also verified the success of GelMA synthesis, where, in comparison with the gelatin spectrum, we observed that the C = O stretching of amide I shifted to 1660 cm^−1^ (Figure 1I), which is due to the overlapping of the stretching signals at 1695 cm^−1^ (C = C band) from methacrylate. A similar result was also observed in the FTIR spectrum of Alg‐MA (Figure S2, Supporting Information), indicating the successful synthesis of the Alg‐MA monomer. Next, the mixture of BPPD nanocomposite and GA was cured under 405 nm visible light to form a nanocomposite hydrogel (**Figure** **2**A). The BPPD nanocomposite together with PDA chains would facilitate interaction with the GA network via both covalent (free‐radical photo‐polymerization) and noncovalent (hydrogen bonds and π–π stacking between catechol groups of PDA chains) forces.

For bone tissue engineering scaffolds, porous structures that promote cell migration and adhesion are essential, so the morphological structure of the as‐prepared hydrogels was examined, as shown in Figure 2B. From the macroscopic photographs, the GA hydrogels were initially opalescent in color, while the BPPD loading did not significantly change the color, probably due to a very small amount of loading. The cross‐sectional scanning electron microscopy (SEM) images showed that all hydrogels exhibited a highly interconnected and porous structure with relatively smooth pore walls, which is similar to that of bone ECM. No obvious BPPD was observed in the pore walls of the hydrogel, probably because of the good interfacial compatibility between the GA matrix and BPPD nanocomposites. Such a porous 3D network structure (e.g., interconnected pores and high porosity) provided an ECM‐mimicking environment (Figure 2C), which was demonstrated to contribute to cell adhesion, migration, and transport of nutrients and metabolic wastes. Micro‐CT reconstruction and parameter analysis showed that all hydrogels had almost the same 3D porous structures and porosity (Figure 2D,E), which holds great potential for facilitating vascular formation and bone regeneration. It is worth noting that the GA/BPPDH hydrogel seemed denser than the other hydrogels, and this phenomenon may be explained by the introduction of PDA increasing the additional crosslinking density in the hydrogel matrix. These results demonstrated that the global structure of the GA hydrogels did not change considerably after BPPD loading, which could be considered as a stable carrier for BPPD.

The material surface hydrophilicity will influence cellular behaviors on the interface between the implant surface and host surrounding tissues. In view of this, the hydrophilicity of all hydrogels was evaluated by measuring their water contact angle. As shown in Figure 2F, the water contact angle was 74.3 ± 3.1° for GA, 61.7 ± 2.5 ± 4.3° for GA/BPPDL, 37.3 ± 2.1° for GA/BPPDM, and 31.3 ± 2.1 for GA/BPPDH, indicating the improved hydrophilicity of these hydrogels after the introduction of BPPD. The excellent hydrophilicity of the GA/BPPD hybrid hydrogels was strongly ascribed to the hydrophilic properties of the PDA and BP components, which was consistent with those of findings reported previously.^[^ ^31^, ^32^ ^]^ Thus, the GA/BPPDM and GA/BPPDH hydrogels showed better wettability than the other hydrogel groups, which were expected to exhibit high performance in favor of cell adhesion, proliferation, growth, spreading, and differentiation.

To achieve successful bone regeneration, the ideal implanted bone materials should possess long‐term structural stability and mechanical support. As shown in Figure 2G, the hybrid hydrogels could maintain their integrity and recover to their original shape without obvious breakage or collapse after repeated compression. The oscillatory rheology of the hydrogels was further tested to investigate their mechanical properties and stability, as shown in Figure 2H. As the oscillation frequency increased, the values of G′ (storage modulus) were consistently greater than G″ (loss modulus), showing good elasticity and mechanical stability of the hydrogel. Meanwhile, the G′ of the hydrogel increased with the addition of BPPD, indicating that the mechanical strength of the hydrogel increased with the increase of the crosslinked network. Notably, increasing the amount of BPPD from 0.5 wt.% to 1 wt.% did not further improve its mechanical properties but rather reduced the elastic modulus. This may be attributed to the reduction of the covalent crosslinking density in the GelMA hydrogel as the amount of BPPD was increased.

The compressive properties of the hydrogels were also investigated, as shown in Figure 2I,J. The compressive stress‐strain curve indicated that the mechanical strength of the hydrogel increased with increasing BPPD concentration, which was due to the improved cross‐linking network. In detail, among all hydrogel groups, the GA hydrogel shows low compressive strength according to the stress‐strain curves. With subsequent BPPD loading, the compressive strength of the hydrogels gradually increased, which was consistent with the rheological results. In particular, the compressive strength of the as‐prepared GA/BPPDM hydrogel (45.1 ± 3.1 MPa) was higher than that of the GA (5.3 ± 1.2 MPa), GA/BPPDL (14.3 ± 1.2 MPa), and GA/BPPDH (26.3 ± 2.6 MPa) hydrogels. The results of the mechanical test demonstrated that the incorporation of BP‐based materials in the hydrogel showed significant improvement in the mechanical strength, which was consistent with previous studies.^[^ ^33^ ^]^ Notably, the enhanced stiffness might regulate cell adhesion, cell growth, and osteogenic differentiation by the enhanced mechanotransduction effect.^[^ ^34^ ^]^ However, increased BPPD concentration may lead to an inhomogeneous distribution in the GA matrix, resulting in the compromised mechanical strength of GA/BPPDH compared with GA/BPPDM. Through rheological and mechanical performance tests, it can be found that the GA/BPPDM hydrogel has a larger energy storage modulus and mechanical strength, so it is more resistant to external deformation and less susceptible to damage.

In addition to desirable structural and mechanical properties, the swelling and degradation profiles of hydrogels play an important role in maintaining the stability of implants and promoting tissue regeneration. As shown in Figure 2K, the swelling of all hydrogels increased rapidly within 60 min and reached equilibrium within 48 h. The swelling ratios of GA, GA/BPPDL, GA/BPPDM, and GA/BPPDH were 1329 ± 50%, 958 ± 36%, 726 ± 40%, and 598 ± 36%, respectively, after 48 h, implying that the swelling ratio of hydrogels decreased with increasing concentrations of BPPD. The declined equilibrium swelling capacities of GA/BPPD may be due to their high crosslinking densities, preventing greater swelling of the hydrogel and buffer diffusion. To observe the in vitro degradation of hydrogels, freeze‐dried samples were collected and weighted after immersion into PBS solution in the presence of lysozyme (100 mg mL^−1^). All hydrogels gradually degraded with prolonged immersion time, and the degradation of GA/BPPD was retarded owing to the increased cross‐linking density in the hydrogel matrix caused by BPPD loading (Figure 2L). The results showed similar trends in the degradation and swelling behavior of the hydrogels. Collectively, GA/BPPD hydrogels with low swelling ratio and slow biodegradation rate are suitable for bone regeneration applications.

### 2.2 Photothermal Performance and Drug Release Behaviors of the Hydrogels

**Figure 3:**
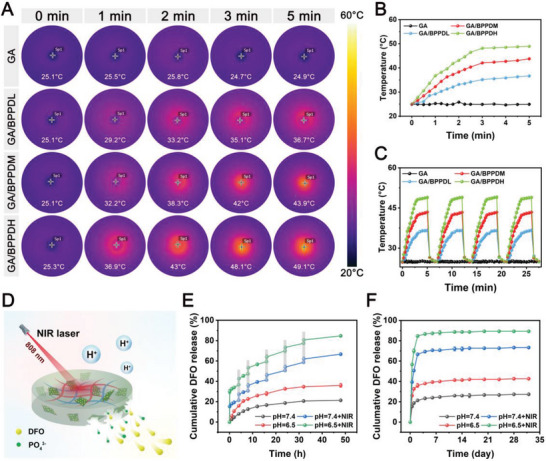
A) Real‐time infrared thermal images and B) photothermal heating curves of the GA/BPPD hydrogel with mild NIR irradiation powers (808 nm, 1 W cm^−2^). C) Photothermal stability of the GA/BPPD hydrogel with four on/off cycles. D) Schematic diagram of the NIR/pH dual‐triggered drug/ion release behavior of the GA/BPPDM hydrogel. E,F) Cumulative release of DFO from the GA/BPPDM hydrogel at different pH values with or without NIR irradiation (808 nm, 1 W cm^−2^). Data are presented as the mean ± SD (*n* = 3).

Recently, photothermal therapy (PTT) has emerged as a promising treatment modality to accelerate bone repair because of its remote controllability, non‐invasive properties, good therapeutic effectiveness, and strong tissue penetration depth.^[^ ^35^ ^]^ Both BP and PDA have been proven to have good photothermal effects.^[^ ^19^ ^]^ To evaluate the photothermal conversion efficiency of GA/BPPD, all hydrogel samples were irradiated with 808 nm NIR light at 1 W cm^−2^. As depicted in **Figure** **3**A, upon NIR irradiation, the temperature of the GA/BPPD hydrogels increased gradually, while the GA hydrogel group showed no obvious temperature variation recorded by an infrared thermogram. After 5 min of NIR irradiation, the temperature was 36.7 ± 0.2 °C for the GA/BPPDL group, 43.8 ± 0.1 °C for the GA/BPPDM group, and 48.9 ± 0.2 °C for the GA/BPPDH group (Figure 3B). Notably, with expending irradiation time, the temperature of GA/BPPDM can eventually reach an equilibrium temperature (≈45 °C), meeting the basic biosafety requirements of mild photothermal biomaterials in promoting tissue regeneration. Moreover, the photothermal stability of the GA/BPPD hydrogel was investigated, as illustrated in Figure 3C. The corresponding results showed that the GA/BPPD hydrogels could be heated and cooled to fixed values without significant attenuation after four cycles of laser on/off, indicating high photostability and great potential to act as NIR‐controlled PTT. Previous studies have found that mild heat stimulation (≈45 °C) can not only promote angiogenesis and osteogenesis but also induce M2 phenotype polarization of macrophages.^[^ ^12^, ^36^ ^]^ Significantly, the temperature of NIR‐triggered efficient mild PTT treatment was usually controlled to be below 45 °C to avoid side effects on normal tissues. In summary, after combining mechanical characterization and the requirement of mild‐temperature PTT (≈45 °C), we selected the GA/BPPDM hydrogel group as a suitable specification for subsequent experiments due to comprehensive consideration.

To realize an appropriate regenerative microenvironment for bone regeneration, it is essential to develop controlled release formulations that can deliver multiple bioactive factors to manipulate cellular functions. The in vitro release kinetics of DFO from GA/BPPD were calculated by UV–vis spectrophotometry at a wavelength of 485 nm. Moreover, the drug loading capacity of DFO was characterized according to the standard calibration curve and is shown in Figure S3 (Supporting Information), with a loading efficiency of DFO reaching 79.8%. As shown in Figure 3D, GA/BPPDM showed typical dual NIR/pH‐dual‐responsive release behavior, and both NIR laser irradiation and the acidic environment could considerably accelerate the release of DFO, showing a similar phenomenon to previous studies.^[^ ^20^, ^37^ ^]^ It is known that the initial physiological characteristics of bone injury are mainly a slightly acidic pH (pH≈6.5), whereas the pH of healthy tissues is 7.3–7.4.^[^ ^6^ ^]^ As such, the pH‐responsive drug release from GA/BPPDM was monitored under neutral (pH = 7.4) and slightly acidic (pH = 6.5) conditions to mimic the healthy and bone injury‐related physiological microenvironments, respectively. The cumulative release of DFO was only ≈21.3% at pH = 7.4 after 48 h, whereas at pH = 6.5, the amount released in 48 h reached ≈36%. Such acid‐accelerated DFO release behavior was ascribed to the pH sensitivity of the PDA layer and protonation of amine groups, which could result in the disruption of π−π interactions between DFO and PDA. As displayed in Figure 3E,F, the release peak of DFO appeared in the first two days and then quickly decayed, indicating that DFO was quickly released within a short time frame (≈7 days). Since bone remodeling requires rapid angiogenesis to provide adequate nutrition delivery, which is conducive to the formation of new bone and the survival of deep bone tissue, the relatively quick release of DFO from hydrogels is favorable for stimulating timely neovascularization and subsequently robust bone formation.

In addition, the NIR‐responsive release behavior of DFO was investigated. Compared with internal pH stimuli, using NIR as external stimuli for drug delivery control exhibits several advantages, such as high tissue penetration, low tissue harm, easy operation, and spatiotemporal precise control of treatment.^[^ ^19^ ^]^ As can be seen from Figure 3E,F, the heat effect generated by NIR laser irradiation obviously affected DFO release. Burst drug release occurred after NIR laser irradiation was applied for 5 min at predetermined time intervals under different pH values. Under the action of the photothermal effect, the final cumulative release percentage of DFO reached ≈66.7% and 84.7% at pH values of 7.4 and 6.5, respectively, during the initial 48 h. In the following release time, the release rate of DFO slowed significantly, and the cumulative release percentage was 73.3% and 89.3% at pH values of 7.4 and 6.5, respectively. These curves presented a burst release in the first 7 days, followed by a slow and plateaued DFO release. The reason for the controlled drug release mode was probably associated with the enhanced diffusion effect under elevated temperature. This result indicated that GA/BPPDM with photothermal function could further trigger DFO release in a thermal‐stimuli manner. Then, the release of PO_4_^3−^ from the GA/BPPDM hydrogel was investigated by ion chromatography. As shown in Figure S4 (Supporting Information), the cumulative release percentage of PO_4_^3−^ from the hydrogel increased over the soaking time, and a relatively fast release of PO_4_^3−^ within 10 days was observed. Although the release rate of PO_4_^3−^ slowed down with an increase in immersion time, the cumulative release curve suggested that PO_4_^3−^ release could continue for up to 28 days in vitro under NIR or without NIR treatment. This sustained release behavior was mainly because the protective effects of the hydrogel matrix and organic coating composed of PDA and DFO synergistically reduced the release of PO_4_^3−^ from the BPPD nanocomposites. A sustainable and slow release of PO_4_^3−^ has been shown to be better for osteogenic differentiation, while the relatively fast release of DFO is preferred for angiogenesis.^[^ ^7^, ^15^ ^]^ These results directly confirmed that the GA/BPPDM hydrogel had both intelligent controlled‐release (DFO) and sustained‐release (PO_4_^3−^) capacities, which made the GA/BPPDM hydrogel as a desirable platform to promote bone regeneration over a long‐term period. From the above results, it was suggested that our nanocomposite hydrogel has a “smart” drug release feature, that is, “switching on” enhanced drug release under both NIR laser irradiation and slightly acidic conditions to enhance bone regeneration efficacy. With the assistance of external NIR photothermal stimuli and bone injury site environmental changes, such as pH, codelivery of DFO/PO_4_^3−^ for synergistic immunomodulation, revascularization and efficient bone regeneration could be achieved. Considering the above rationale, the smart‐responsive GA/BPPDM hydrogels will be promising in the applications of bone defect treatment and even other tissue regeneration.

### 2.3 In Vitro Evaluation of Cytocompatibility

**Figure 4:**
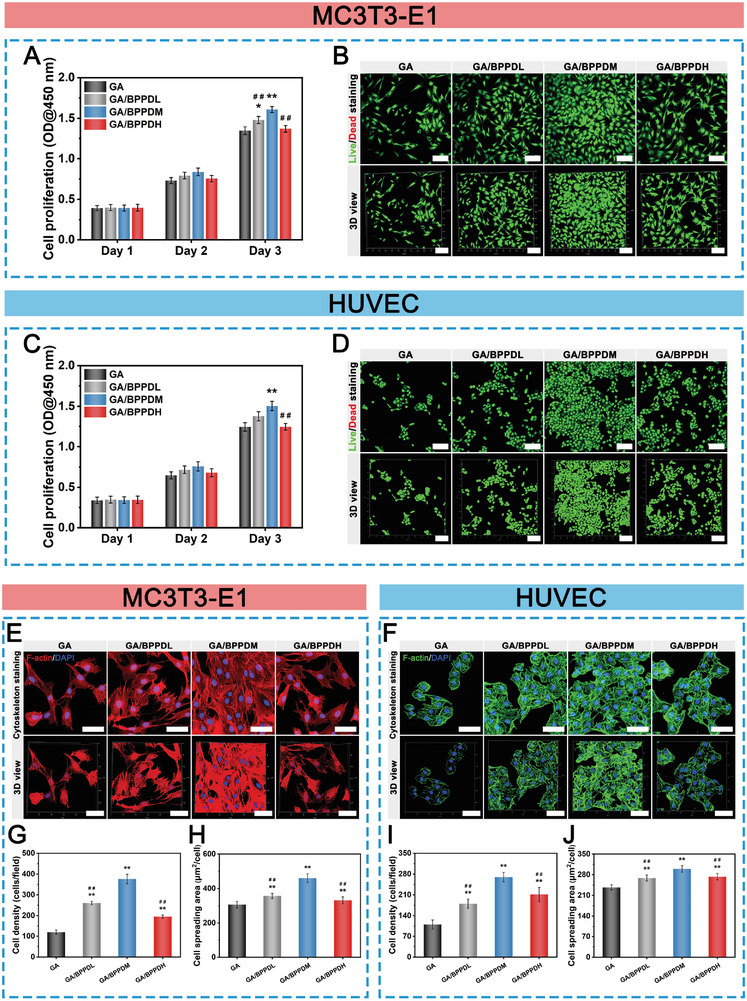
A) CCK‐8 assay and B) live/dead staining of MC3T3‐E1 cells cultured on the hydrogels. C) CCK‐8 assay and D) live/dead staining of HUVECs cultured on the hydrogels. Cytoskeleton fluorescence staining of F‐actin and DAPI in E) MC3T3‐E1 cells and F) HUVECs cultured on the hydrogels for 3 days. Quantitative analysis of G) cell density and H) cell spreading area of MC3T3‐E1 cells. Quantitative analysis of I) cell density and J) cell spreading area of HUVECs. Scale bar: 200 µm (B,D), 75 µm (E,F). Data are presented as the mean ± SD (*n* = 3). *p < 0.05 and **p < 0.01 indicate significant differences compared with the GA group. ^#^ *p* < 0.05 and ^# #^ *p* < 0.01 indicate significant differences compared with the GA/BPPDM group.

In the following experiments, both MC3T3‐E1 cells and HUVECs were used to evaluate the influence of the nanocomposite hydrogels on cell viability and proliferation, as they are the major and important cell sources for bone formation and vascular regeneration.^[^ ^38^, ^39^ ^]^ The results of the CCK‐8 assay revealed that the OD values of the four hydrogel groups increased with the culture time prolonging (**Figure** **4**A,C), indicating that the cells maintained good viability and proliferation ability. More specifically, cell proliferation on day 1 was similar for all groups and the cells co‐cultured on the hydrogels proliferated over time. After 2 days of co‐incubation, compared to the GA hydrogel, the hydrogels loaded with active BPPD nanocomposites showed a slightly improved cell proliferation rate, although there was no obvious difference among the four groups of GA, GA/BPPDL, GA/BPPDM, and GA/BPPDH. When it came to 3 days, the highest cell proliferation rate was found in GA/BPPDM, followed by GA/BPPDL and GA/BPPDH, then GA, revealing that the hydrogel group loaded with BPPD, especially 0.5 wt.%, was more beneficial for promoting cell proliferation. The excellent cell affinity of these hydrogels was also confirmed by live/dead staining assay, as shown in Figure 4B,D. After co‐culturing for 3 days, almost all MC3T3‐E1 cells and HUVECs were alive (green fluorescence) in the GA, GA/BPPDL, GA/BPPDM, and GA/BPPDH hydrogels, with only a few dead cells present (red fluorescence), indicating that the composite hydrogels could support cell survival and growth (viability > 90%). According to the quantitative results, all hydrogels had excellent cytocompatibility for both MC3T3‐E1 cells and HUVECs, and the existence of BPPD substantially increased the cell density and the percentage of living cells (Figure 4G,I), improving the bioactivity of the hydrogel. Notably, significantly promoted proliferation of cells was observed in the GA/BPPDM hydrogel group compared with GA/BPPDL and GA/BPPDH, which was in accordance with the results of the CCK‐8 analysis. Both GelMA and Alg‐MA have high biocompatibility and mimic the chemical properties of the ECM; meanwhile, BP and PDA have shown excellent biocompatibility in previously reported studies.^[^ ^17^, ^29^ ^]^ The enhanced cell proliferation was likely due to phosphate and DFO release into the culture medium, which was consistent with literature reports.^[^ ^40^ ^]^ However, with a concentration higher than 0.5 wt.%, no obvious enhancement was observed, mostly because higher concentrations of phosphate and DFO disturbed normal cell activity. This implied that at an appropriate level, BPPD nanomaterials might promote cell growth, which can also explain why cells on GA/BPPDM are well proliferated and widely distributed.

The adhesion and spreading morphologies of the cells were further observed by cytoskeleton staining. After being co‐cultured for 3 days, the cytoskeletal morphology of MC3T3‐E1 cells revealed that the cells in the GA group had only a few pseudopodia and had not completely spread on the hydrogel. In contrast, the cells adhered to the GA/BPPD hydrogels exhibited better stretched morphology with well‐developed cell cytoskeletons and clusters, extending numerous branchy filamentous pseudopods and tightly interleaving with each other (Figure 4E; Figure S5A, Supporting Information). In particular, the cells on the GA/BPPDM hydrogel spread well with a typical elongated‐spindle and osteoblastic‐like morphology, indicating a favorable growth status, which may be attributed to its optimal BPPD concentration and hydrophilicity. Additionally, we also observed increased numbers of cell colonies on GA/BPPDM samples compared to pure GA, which allowed for more cell attachment and spreading, revealing the positive effects of BPPD on improving cell migration and proliferation, which is consistent with the results of cell toxicity and proliferation assays. Recent studies have found that cell morphology plays a vital role in regulating the phenotype of cells, and an elongated spindle‐shaped morphology has been found to be tightly correlated with the adhesion, proliferation, and differentiation of osteoblast‐related cells and endothelial cells.^[^ ^41^, ^42^ ^]^ The enlarged confocal laser scanning microscopy (CLSM) images further verified the good cell adhesion and spreading behaviors on the GA/BPPDM hydrogel (Figure S5A, Supporting Information), as evidenced by the stretched filopodia and cytoskeletal rearrangement. According to the CLSM observation (Figure 4E) and quantitative results (Figure 4G,H), more cells adhered to the GA/BPPDM hydrogel not only exhibited the highest spreading area, but the actin filaments that make up the cytoskeleton were also highly expressed, implying a strong interaction between cells and the GA/BPPDM hydrogel. Concomitantly, similar results were also detected in HUVECs grown on the hydrogels, in which the HUVECs grown on the GA/BPPDM hydrogel presented well‐stretched morphology and favorable proliferation (Figure 4F,I,J; Figure S5B, Supporting Information). These encouraging results suggested that the incorporation of moderate BPPD contributed to the adhesion behavior of MC3T3‐E1 cells and HUVECs due to the nature of each raw material, including PDA, DFO, and BP nanosheets, which demonstrated good cell affinity and improved cell crawling and adhesion properties.^[^ ^7^, ^15^, ^43^ ^]^ With high bioactivity and hydrophilicity, BPPD nanomaterials might serve as cell adhesion sites that promote cell growth, spreading, and differentiation. Overall, our data showed that all fabricated GA/BPPD nanocomposite hydrogels, especially GA/BPPDM, possessed remarkably positive effects on MC3T3‐E1 cell and HUVEC proliferation, survival, and growth. Considering the vital role of cell proliferation in cell differentiation and subsequent tissue formation, we chose the GA/BPPDM hydrogel to further verify its osteogenesis, angiogenesis, and immunomodulatory capability in vivo and in vitro.

### 2.4 In Vitro and In Vivo Immunomodulatory Properties

**Figure 5:**
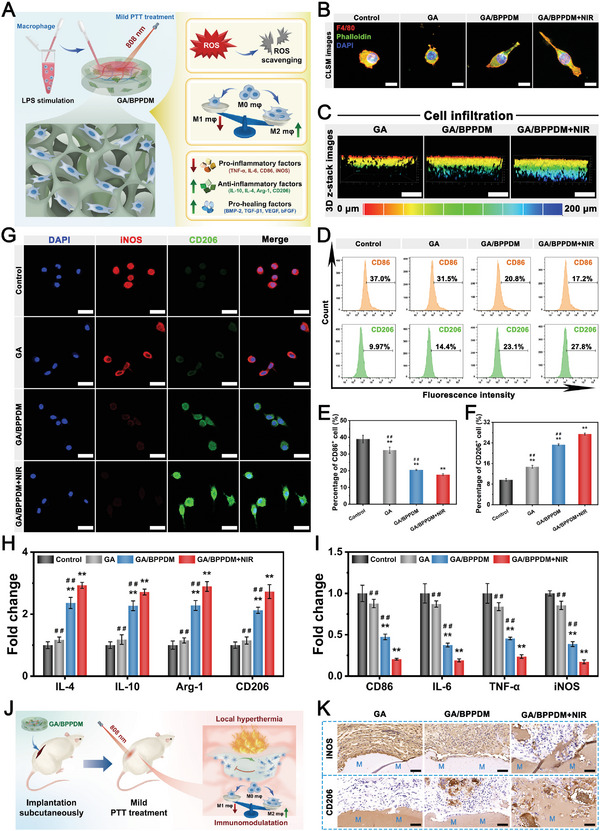
A) Schematic diagram of exploring the effect of GA/BPPDM hydrogels combined with NIR irradiation on ROS scavenging and immunomodulation. B) Immunofluorescence staining of F4/80 and F‐actin in RAW264.7 macrophages after different treatments. C) 3D z‐stack images of RAW264.7 macrophage penetration depth after incubation for 4 days. D) Flow cytometry analysis and E,F) quantification of CD86 and CD206 expression in RAW264.7 macrophages. G) Immunofluorescence staining of iNOS and CD206 in RAW264.7 macrophages. H,I) Relative mRNA expression of inflammation‐related genes in RAW264.7 macrophages. J,K) Immunohistochemical staining of iNOS and CD206 in different hydrogels on day 7 after implantation in the rat subcutaneous model (M: material residue). Scale bar: 5 µm (B), 200 µm (C), 20 µm (G), 50 µm (K). Data are presented as the mean ± SD (*n* = 3). *p < 0.05 and **p < 0.01 indicate significant differences compared with the control group. ^#^ *p* < 0.05 and ^# #^ *p* < 0.01 indicate significant differences compared with the GA/BPPDM+NIR group.

Although multiple immune cell types are involved in the immune response and microenvironment regulation, macrophages have been demonstrated to play a prominent role in tissue regeneration and remodeling. Following bone injury, macrophages predominate as proinflammatory phenotypes (M1) at the inflammation stage and contribute to the characteristics of high ROS in response to local inflammatory signals.^[^ ^44^ ^]^ Unfortunately, excessive inflammatory response‐induced overproduction of ROS in the bone defect region has a detrimental impact on bone regeneration. Increased levels of ROS in the bone defect can not only cause cell death in osteoblast precursor cells and mature osteoblasts but also reduce the expression of osteogenic markers and mineralization, leading to prolonged and unhealed bone injury.^[^ ^6^ ^]^ Therefore, the development of functional biomaterials with excellent immunomodulatory effects and ROS‐scavenging capacity is of great significance to promote the regeneration of bone tissue (**Figure** **5**A). In this work, RAW264.7 cells were selected as the model of macrophages and then treated with LPS (a component of Gram‐negative bacterial cell walls) to imitate acute inflammatory responses and induce macrophages to the M1 phenotype, thus leading to the production of numerous free radicals (i.e., ROS) and long‐term inflammation. Macrophages were co‐cultured with different hydrogels, and the macrophage response was then assessed via immunofluorescence staining and flow cytometry. As shown in Figure S6A (Supporting Information), the highest expression level of ROS was observed in the control group exposed to LPS, suggesting that cellular oxidative stress was successfully induced. Interestingly, both GA/BPPDM and GA/BPPDM+NIR effectively inhibited LPS‐provoked ROS generation, as evidenced by the decreased 2′,7′‐dichlorofluorescein diacetate (DCFH‐DA) fluorescent signals and the shift of its relative fluorescence intensity to the left (Figure S6B, Supporting Information). More importantly, cells treated with GA/BPPDM plus NIR irradiation exerted the most significant inhibitory effects on total ROS generation, showing negligible green fluorescence, which indicated a strong ability to protect cells from ROS‐induced oxidative damage. Thus, these results demonstrated that GA/BPPDM combined with mild photothermal treatment through NIR irradiation could reduce the excessive oxidative stress of cells induced by the inflammation‐related response, showing protective effect on cell function. Studies have shown that in addition to a notable promoting effect on osteogenesis, BP has a strong ROS‐scavenging capability and high bioactivity, showing great prospects for application in promoting tissue repair and regeneration.^[^ ^45^ ^]^ Additionally, owing to the presence of abundant catechol groups, PDA could efficiently eliminate intracellular ROS and thus exhibit anti‐inflammatory ability by regulating the proportion of M1/M2 macrophages,^[^ ^26^, ^46^ ^]^ and the anti‐inflammatory effect of DFO has been reported more frequently.^[^ ^22^ ^]^ The anti‐inflammatory effect of DFO was achieved through its excellent iron‐chelating ability, which enabled it to scavenge free radicals and ROS generated after acute inflammation. Furthermore, the mild photothermal effect (41 ± 1 °C) triggered by NIR irradiation could further enhance the radical scavenging ability of the hybrid hydrogel, which may be related to accelerated disassembly of BPPD, thus facilitating adequate contact between reductive components and free radical detection reagents, consistent with the results of previous work.^[^ ^47^ ^]^ On the basis of previously published studies, our current findings supported the excellent antioxidant ability of the GA/BPPDM hydrogel system because of the combined effects of bioactive components (BP, PDA, and DFO) and mild photothermal activity upon NIR irradiation.

Accumulating data show that M1‐type macrophages aggravate inflammation, while M2‐type macrophages with anti‐inflammatory effects can ameliorate the local inflammatory microenvironment and promote tissue regeneration.^[^ ^28^ ^]^ Thus, guiding the polarization of macrophages toward the regenerative M2 phenotype is more likely to achieve immune‐mediated bone regeneration. To evaluate the effect of the GA/BPPDM hydrogel platform on macrophage reprogramming, we first collected RAW264.7 cells and investigated the morphological changes by cytoskeleton staining. As shown in Figure 5B, macrophages cultured on GA/BPPDM hydrogels under NIR laser irradiation appeared as elongated and flattened spindle‐like cells, which are the morphological characteristics of M2 macrophages. Subsequently, the effects of the hydrogels with or without NIR radiation on the 3D migration of macrophages were investigated by cytoskeleton staining. Recent studies have reported that macrophage infiltration is the initial and critical step in the process of regeneration, while promoting macrophage infiltration accelerates hard callus formation and ossification.^[^ ^48^ ^]^ As displayed in Figure 5C, 3D‐reconstructed CLSM images demonstrated that the macrophages not only adhered to the surface of the hydrogels but also gradually infiltrated into the 3D network of the hydrogel matrix. Importantly, the cells treated with GA/BPPDM+NIR exhibited the strongest migration and penetration capabilities, followed by the GA/BPPDM group. In sharp contrast, cell infiltration was almost invisible inside the GA hydrogel, in which cells mainly grew on the top surface of the hydrogel.

To validate the phenotypes of polarized macrophages after treatment, the M1 and M2 phenotypes of macrophages were labeled with CD86 and CD206, respectively, and then detected by flow cytometry. As shown in Figure 5D, a significant increase of CD86 (an M1 marker) expression was observed in RAW264.7 cells after treatment with LPS, while the expression of CD86 in the GA, GA/BPPDM and GA/BPPDM+NIR groups was reduced, indicating the activation of macrophage polarization toward the M2 phenotype. Specifically, higher levels of CD206 (an M2 marker) expression were observed in the GA/BPPDM group than in the LPS and GA groups, and this effect was even more pronounced after periodic and appropriate NIR irradiation. According to the statistical results in Figure 5E,F, significantly higher ratios of M2 macrophages (CD206^+^ cells) and decreased M1 macrophages (CD86^+^ cells) were observed in the GA/BPPDM+NIR group with better immunomodulatory ability. These results demonstrated that the combined therapy of GA/BPPDM plus mild PTT treatment was beneficial for switching the macrophage phenotype from M1 toward M2, leading to the creation of an anti‐inflammatory microenvironment. Immunofluorescence staining further confirmed that GA/BPPDM plus mild photothermal effect was more conducive to macrophage M2 polarization than GA and GA/BPPDM alone (Figure 5G). The quantitative fluorescence intensity also showed that the GA/BPPDM+NIR group presented higher CD206^+^ expression and lower iNOS^+^ expression (Figure S7, Supporting Information). According to our in vitro results, GA/BPPDM combined with mild thermal stimulation at 41 ± 1 °C could promote M2 polarization of macrophages and inhibit the expression of M1 macrophages under NIR irradiation conditions, showing huge potential to shorten the inflammation phase and shift it into the proliferation phase during bone regeneration. To further reveal the role of GA/BPPDM and NIR treatment in the immune response, the expression of a series of inflammatory and pro‐healing cytokines was evaluated in the cell supernatant by ELISA. As expected, GA/BPPDM was associated with increased secretion of anti‐inflammatory IL‐10 and IL‐4 and decreased secretion of proinflammatory TNF‐α and IL‐6 under thermal stimulation provided by NIR irradiation (Figure S8, Supporting Information). More significantly, compared with the other groups, the GA/BPPDM+NIR group secreted much more pro‐osteogenic (BMP‐2, TGF‐β1) and pro‐angiogenic (VEGF, bFGF) factors, followed by the GA/BPPDM and GA groups (Figure S9, Supporting Information). Previous studies have found that M2‐type macrophages can participate in bone regeneration by regulating the release of growth factors (BMP‐2, TGF‐β1, VEGF, and bFGF) and paracrine signals.^[^ ^3^ ^]^ The GA/BPPDM+NIR hydrogel system is able to polarize macrophages to an anti‐inflammatory M2 phenotype, which is consistent with the results of flow cytometry, resulting in the release of pro‐regenerative factors associated with osteogenesis and angiogenesis. Likewise, both real‐time polymerase chain reaction (qRT‐PCR) and Western blot results confirmed that M2 phenotypic markers, such as IL‐4, IL‐10, Arg‐1, and CD206, were significantly upregulated in the GA/BPPDM+NIR group (Figure 5H; Figure S10, Supporting Information). On the contrary, the expression of M1 phenotypic markers, such as CD86, IL‐6, TNF‐α, and iNOS, was relatively lower in the GA/BPPDM+NIR group than in the GA/BPPDM group (Figure 5I; Figure S10, Supporting Information). These results suggested that the GA/BPPDM hydrogel system together with NIR‐triggered drug release had the potential to synergistically alleviate the inflammatory reaction and induce tissue regeneration through the transition of M1‐to‐M2 macrophage polarization.

Previous studies have shown that mild hyperthermia triggered by NIR irradiation could induce an increase in anti‐inflammatory cytokine secretion, ROS scavenging, and the transformation of the M1‐M2 phenotype of macrophages via activation of the PI3K/Akt1 signaling pathway, leading to a favorable regenerative microenvironment for tissue regeneration.^[^ ^12^, ^49^ ^]^ The role of the PI3K/Akt1 signaling pathway in the regulation of macrophage polarization has been well studied, and PI3K is an upstream regulator of protein kinase B (Akt) and has been demonstrated to modulate the phenotype of M2 macrophages.^[^ ^50^ ^]^ Given that, the PI3K/Akt1 signaling pathway was subsequently verified via Western blot analysis. As shown in Figure S11 (Supporting Information), the GA/BPPDM+NIR group remarkably enhanced the PI3K, Akt1, and p‐Akt1 protein expression of RAW264.7 cells, indicating that PI3K/Akt1 signaling is activated, which may be important to promote cascading macrophage M2 polarization. These results further suggested that the photothermal GA/BPPDM hydrogel system may therapeutically alleviate inflammation and induce the polarization of macrophages toward the M2 phenotype by activating the PI3K/Akt1 signaling pathway to downregulate the expression of inflammatory cytokines.

To further identify the immunomodulatory ability during the early stage of implantation, we evaluated the macrophage phenotypes in subcutaneously embedded tissue sections on day 7 as mentioned above (Figure 5J). Real‐time infrared thermal images were captured after irradiating the implanted hydrogel samples with an 808 nm NIR laser. Consistent with the in vitro polarization of macrophages, immunohistochemical staining confirmed that the GA/BPPDM+NIR group was more conducive to macrophage M2 polarization than the GA/BPPDM group (Figure 5K). Quantitative analysis showed higher CD206^+^ expression and lower iNOS^+^ expression in the GA/BPPDM+NIR group than in the GA/BPPDM group (Figure S12, Supporting Information). The proteins of the cells in the subcutaneously embedded hydrogel were extracted and processed for ELISA measurement. The GA/BPPDM hydrogel with NIR stimulation showed higher expression of the anti‐inflammatory factor IL‐10 and lower expression of the proinflammatory factor TNF‐α (Figure S13, Supporting Information), which is also consistent with the in vitro tests. The GA/BPPDM photothermal therapeutic platform effectively alleviated inflammation and altered the secretion of cytokines in the microenvironment through immunomodulation compared to the GA/BPPDM and GA groups alone. Based on these in vitro and in vivo results, it is summarized that the GA/BPPDM hydrogel could modulate macrophage polarization and promote anti‐inflammatory processes under on‐demand NIR irradiation, thus shortening the inflammatory phase.

### 2.5 In Vitro and In Vivo Angiogenesis Assay

**Figure 6:**
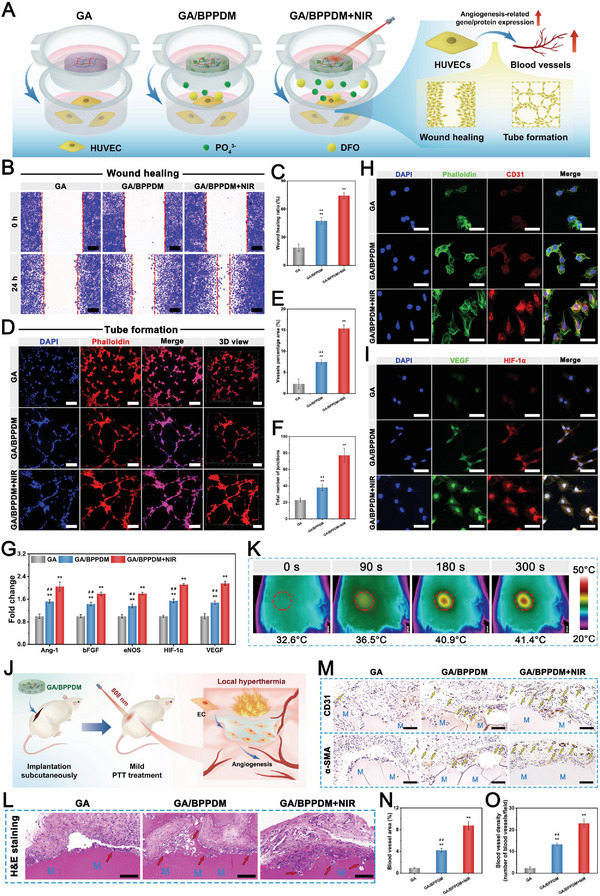
A) Schematic diagram of exploring the effect of GA/BPPDM hydrogels combined with NIR irradiation on the angiogenic differentiation of HUVECs. B) Crystal violet staining and C) quantitative analysis of the scratch wound healing assay. D) CLSM images of the tube formation assay and E,F) quantitative analysis of tube formation, including the average vessel percentage area and the total number of junctions. G) Relative mRNA expression of angiogenesis‐related genes in HUVECs, including Ang‐1, bFGF, eNOS, HIF‐1α, and VEGF. H,I) Immunofluorescence staining of CD31, VEGF, and HIF‐1α in HUVECs. J) Schematic diagram of the rat subcutaneous implantation model and the process of blood vessel formation. K) Real‐time infrared thermal images of rats implanted with GA/BPPDM hydrogel under NIR irradiation for 5 min (1 W cm^−2^, 808 nm). L) H&E staining and M) immunohistochemical staining for CD31 and α‐SMA at 14 days after implantation (M: material residue, red arrow: infiltration of host cells, yellow arrow: newly formed blood vessels). N,O) Quantitative analysis of the CD31‐positive expression area and microvessel density. Scale bar: 200 µm (B, D), 50 µm (H, I), 100 µm (L,M). Data are presented as the mean ± SD (*n* = 3). *p < 0.05 and **p < 0.01 indicate significant differences compared with the GA group. ^#^ *p* < 0.05 and ^# #^ *p* < 0.01 indicate significant differences compared with the GA/BPPDM+NIR group.

Accumulating evidence has well‐established that angiogenesis is essential for the reconstruction processes of bone healing, which helps to promote new bone formation by accelerating the transportation of nutrients, signaling molecules, and so on.^[^ ^39^, ^51^ ^]^ In view of this, satisfactory pro‐angiogenic activities are required for advanced functional biomaterials to satisfy the demands for bone healing. To evaluate the potential effects of NIR‐triggered drug release of the hydrogels on angiogenesis, HUVECs cultured with the hydrogels with or without NIR irradiation were subjected to angiogenic differentiation assay (**Figure** **6**A). The effect of the hydrogel on HUVEC migration was first evaluated by wound healing experiments. Cell migration appeared in all groups and was much better in the GA/BPPDM and GA/BPPDM+NIR groups than in the GA group (Figure 6B). The GA/BPPDM+NIR group displayed better wound closure at 24 h, in which the scratch was almost closed, followed by the GA/BPPDM group. The quantitative analysis further indicated better wound closure in the GA/BPPDM+NIR groups than in the GA/BPPDM and GA groups at 24 h (Figure 6C), which was mainly ascribed to the accumulated release of DFO triggered by periodic NIR irradiation. In vitro Transwell migration assay was also performed to investigate the potential of the hydrogel system to induce HUVEC migration. As shown in Figure S14 (Supporting Information), both the GA/BPPDM and GA/BPPDM+NIR groups could exert a chemotaxis effect on recruiting more HUVECs than the GA group. Additionally, the migration ability was considerably improved by the GA/BPPDM hydrogel upon NIR irradiation, as indicated by the highest number of transmembrane cells observed in the GA/BPPDM+NIR group. Here, the wound healing and Transwell migration assays revealed better migration ability for HUVECs in the GA/BPPDM group, but the promotive effect was not as strong as that of the GA/BPPDM+NIR group. Collectively, these results indicated that the migration ability of HUVECs was effectively improved by GA/BPPDM together with on‐demand NIR irradiation, which was also beneficial for vascular regeneration and bone formation.

The in vitro tube formation assay was further conducted to evaluate the pro‐angiogenic activity of the prepared hydrogels using Matrigel because angiogenesis is a key factor in bone healing, and the ability to promote angiogenesis can accelerate the repair process. As shown in Figure 6D, little tubule formation was observed in the GA group, suggesting that the vasculogenic ability of the cells was limited. As expected, the formation of tubular frameworks was detected in the GA/BPPDM and GA/BPPDM+NIR groups; however, compared with the GA/BPPDM group, more mature and intact tubular structures as well as a higher density of cell junctions were observed in the GA/BPPDM+NIR group. In terms of the quantitative analysis, both the vessel percentage area and total number of junctions were significantly increased in the GA/BPPDM+NIR group, followed by the GA/BPPDM group (Figure 6E,F), indicating that enhanced vessel formation likely occurred because of the cumulative effect of sustainedly released DFO from GA/BPPDM with the assistance of NIR irradiation. The rapid establishment of vessel networks can promote the recruitment of nutrients and related growth factors at the defect area, thereby accelerating the repair process. These results indicated that the outstanding pro‐angiogenic potential of the GA/BPPDM+NIR group was mainly due to continuous NIR‐triggered DFO release, which can effectively induce cell migration and tubule formation in vitro, benefiting the repair and angiogenesis of impaired tissues.

Vascularization during bone healing is known to be regulated by growth factors such as VEGF and bFGF and other downstream angiogenic molecules, including eNOS and HIF‐1α expressed by endothelial cells.^[^ ^52^ ^]^ In view of this, the expression of angiogenesis‐related factors in HUVECs was further studied by qRT‐PCR analysis. As expected, HUVECs in the GA/BPPDM+NIR group owned the highest expression levels of Ang‐1, bFGF, eNOS, HIF‐1α, and VEGF (Figure 6G); this was because of the continuous accumulation of DFO from the GA/BPPDM hydrogel with the help of NIR irradiation. It has been verified that DFO is beneficial for the stimulation of angiogenesis. Meanwhile, DFO can stabilize HIF‐1α expression, followed by upregulating the expression of angiogenic factors such as VEGF.^[^ ^40^ ^]^ As for the GA/BPPDM group, due to the incorporation of BPPD, a significant promotion effect on angiogenic activity was demonstrated. Next, the angiogenic capacity of different hydrogels was further verified by immunofluorescence staining of CD31, VEGF, and HIF‐1α. After 7 days of co‐incubation, the protein expression of CD31 was significantly increased in both the GA/BPPDM and GA/BPPDM+NIR groups, especially in the GA/BPPDM+NIR group, in which the fluorescence signal of CD31 displayed the highest expression level (Figure 6H), suggesting enhanced vascularization. Unsurprisingly, the results of VEGF and HIF‐1α protein expression showed the same tendency that the GA/BPPDM+NIR groups exhibited the most significant expression of VEGF and HIF‐1α protein markers, followed by the GA/BPPDM and GA groups (Figure 6I). The quantitative results of fluorescence intensity also demonstrated the most significant expression of angiogenic protein markers in the GA/BPPDM+NIR group (Figure S15, Supporting Information), indicating enhanced angiogenic activity. From the above results, it was concluded that the GA/BPPDM+NIR group might activate the HIF‐1α pathway and promote the expression of angiogenesis‐related genes/proteins, including Ang‐1, bFGF, eNOS, and VEGF, in HUVECs, which led to the rapid angiogenesis process in vitro.

In bone tissue engineering, early blood vessel formation can provide sufficient nutrient supply and accelerate new bone formation. To evaluate the impacts of hydrogels on the angiogenesis process in vivo, samples were implanted subcutaneously into the backs of rats to observe new blood vessel formation (Figure 6J). During NIR laser irradiation, the temperature changes and corresponding thermal images of the implanted site were recorded by an infrared thermograph, as shown in Figure 6K. After 7 days of implantation, the tissues surrounding the implanted hydrogels were subjected to histological observation. The results of hematoxylin and eosin (H&E) staining showed that quite a lot of surrounding tissue cells could be detected around and within the GA/BPPDM+NIR group (Figure 6L), implying that the GA/BPPDM hydrogel and mild heat stimulation contributed to the fast ingrowth of the surrounding tissues (red arrows), especially to timely vascular growth, which is essential for tissue remodeling. Owing to the biodegradable hydrogel matrix and bioactive BPPD nanomaterial as well as mild heat stimulation, the cells could migrate into the hydrogels rapidly after in vivo implantation. As a result, the GA/BPPDM+NIR group exhibited excellent tissue integration. The in vivo angiogenic abilities of the implanted hydrogels were further investigated by immunohistochemical analyses of CD31 and α‐SMA, as shown in Figure 6M. It is obviously found that there were a greater number of newly formed CD31^+^ and α‐SMA^+^ blood vessels (yellow arrows) around the GA/BPPDM and GA/BPPDM+NIR groups than around the GA group, implying a higher vascular regeneration improvement. The reason might be that the release of DFO in the early stage was demonstrated to improve the angiogenic activity of the hydrogel and promote angiogenesis in vivo, consistent with previously reported studies.^[^ ^53^ ^]^ Significantly, the expression of CD31 and α‐SMA in the GA/BPPDM+NIR group was the highest among the three groups, which was ascribed to the ability of the on‐demand NIR‐assisted mild heat stimulation to promote adhesion, migration, and angiogenesis as well as induce fast DFO release to a greater extent than the hydrogel without NIR treatment. Accordingly, the quantitative results further verified the formation and quantity of blood vessels in the different groups, supporting the robust stimulation of angiogenesis in the initial inflammatory phase by the GA/BPPDM+NIR group (Figure 6N,O), which was consistent with the in vitro results. The above evidence showed that GA/BPPDM could effectively induce the formation of microvessels, and on‐demand NIR irradiation could further accelerate neovascularization due to the synergetic effect of mild heat stimulation and NIR‐triggered DFO release.

### 2.6 In Vitro Evaluation of Osteogenic Differentiation

The ability to recruit endogenous cells from surrounding areas is critical for initiating efficient vessel and bone regeneration. To investigate the effect of the as‐prepared hydrogel platform on recruiting osteoblast precursor cells, a Transwell migration assay was conducted with or without NIR irradiation. As shown in Figure S16 (Supporting Information), with burst release of PO_4_^3‐^ and DFO from the hydrogels upon NIR irradiation, the GA/BPPDM+NIR group significantly improved the recruitment of MC3TE‐E1 cells in vitro. After being co‐cultured for 24 h, the cell numbers recruited by the GA/BPPDM+NIR group were 3.8‐fold and 1.8‐fold that of the GA/BPPDM and GA groups, respectively, demonstrating that BPPD loading and NIR treatment efficiently enhanced MC3TE‐E1 cell recruitment. Previous studies have demonstrated that BP‐incorporated biomaterials display a positive effect on osteogenesis by modulating osteogenic cytokine secretion to recruit osteoblasts and promote osteogenic activity.^[^ ^31^, ^54^ ^]^ Meanwhile, DFO can be rapidly released from the GA/BPPDM hydrogel with the assistance of NIR irradiation and synergize with BP to further promote the proliferation, migration and osteogenic differentiation of cells. This suggested that the NIR‐induced photothermal effect could trigger the release of large amounts of drug/ion quickly to promote cell migration, as evidenced by the results of the release behaviors in vitro.

**Figure 7:**
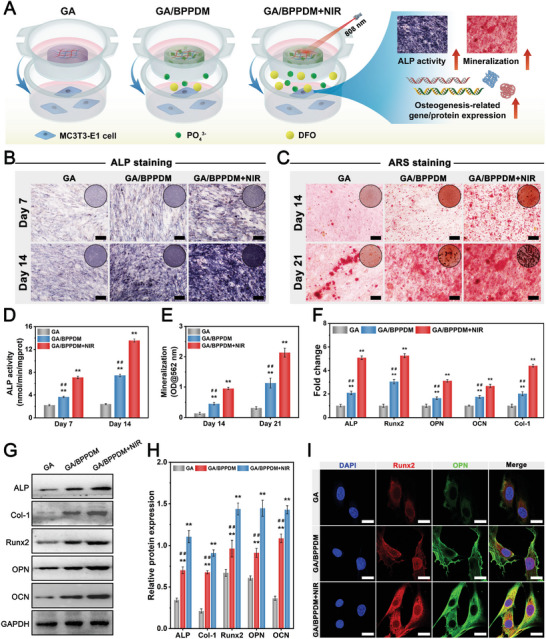
A) Schematic diagram of exploring the effect of GA/BPPDM hydrogels combined with NIR irradiation on the osteogenic differentiation of MC3T3‐E1 cells. Macroscopic and microscopic images of B) ALP staining and C) ARS staining of MC3T3‐E1 cells. Quantitative analysis of D) ALP activity and E) ARS staining. F) Relative mRNA expression of osteogenesis‐related genes in MC3T3‐E1 cells, including ALP, Runx2, Col‐1, OPN, and OCN. G,H) Western blot analysis and quantification of osteogenic protein expression, including ALP, Runx2, Col‐1, OPN, and OCN. I) Immunofluorescence staining of Runx2 and OPN in MC3T3‐E1 cells. Scale bar: 200 µm (B, C), 20 µm (I). Data are presented as the mean ± SD (*n* = 3). *p < 0.05 and **p < 0.01 indicate significant differences compared with the GA group. ^#^ *p* < 0.05 and ^# #^ *p* < 0.01 indicate significant differences compared with the GA/BPPDM+NIR group.

We next assessed the effect of the hydrogel platform on the osteogenic differentiation of MC3TE‐E1 cells. A cell co‐culture system was established using a Transwell device, in which the hydrogels were placed in the upper chamber and MC3TE‐E1 cells in the lower chamber under NIR laser irradiation (**Figure** **7**A). As a hallmark of osteogenic differentiation in the early stage, alkaline phosphatase (ALP) activity was first evaluated by qualitative and quantitative measurements in vitro. As illustrated in Figure 7B, the GA/BPPDM+NIR group exhibited the deepest ALP staining, followed by the GA/BPPDM and GA groups, which confirmed that the GA/BPPDM hydrogel with NIR‐induced PTT treatment promoted the early osteogenic differentiation of MC3TE‐E1 cells. Correspondingly, through the quantitative analysis of ALP activity, both the GA/BPPDM and GA/BPPDM+NIR groups caused a significant increase in ALP production compared with the GA group (Figure 7D). In particular, the ALP activities in the GA/BPPDM+NIR group were significantly higher than those in the other groups on days 7 and 14, demonstrating the synergistic effects of BPPD and NIR‐triggered drug/ion release on inducing osteogenic differentiation in vitro.

The deposition of calcium minerals, as an important indicator of the later stage of osteogenic differentiation, was evaluated by Alizarin red S (ARS) staining. Consistent with the results of ALP activity, both macroscopic and microscopic images showed that the GA/BPPDM+NIR group presented the highest amounts of bone‐mineralized nodules with an enhanced degree of positive staining among all groups (Figure 7C), indicating better osteogenic potential. In a previous study, Shao et al. found that irradiation with NIR light not only accelerated the degradation of BP‐incorporated hydrogels into PO_4_^3−^, but also enhanced the biological activity to facilitate the reaction between PO_4_^3−^ and Ca^2+^, thus promoting bone regeneration through in situ biomineralization.^[^ ^55^ ^]^ In line with the qualitative results, higher levels of mineral matrix formation were detected in the GA/BPPDM and GA/BPPDM+NIR groups, especially in the GA/BPPDM+NIR group (Figure 7E). To further assess the in vitro osteogenic potential of the photothermal hydrogel platform, rat BMSCs were also selected for this study because they are the major and important cell source for bone repair and regeneration (Figure S17A, Supporting Information). Unsurprisingly, the GA/BPPDM+NIR group showed the highest ALP activity and mineralized nodule formation compared with any other group, followed by the GA/BPPDM and GA groups (Figure S17B–E, Supporting Information). These data strongly implied that the incorporation of BPPD could promote osteogenic differentiation in vitro, and NIR‐triggered PO_4_^3‐^ and DFO release further exerted a potent synergistic effect on osteogenesis. Thus, the GA/BPPDM+NIR group could promote osteogenic differentiation and accelerate mineralized matrix formation in both MC3TE‐E1 cells and BMSCs.

It has been reported that Col‐1, OPN, and OCN are important components of the ECM of bone, and Runx2 is a critical transcription factor regulating the expression of osteogenesis‐related genes.^[^ ^32^ ^]^ Therefore, the effects of GA/BPPDM and NIR‐triggered DFO on the osteogenic differentiation of both MC3T3‐E1 cells and BMSCs were investigated through genetic‐ and protein‐level analyses. As illustrated in Figure 7F–H and Figure S17F (Supporting Information), the expression levels of ALP, Runx2, Col‐1, OPN, and OCN substantially increased in the GA/BPPDM and GA/BPPDM+NIR groups compared with the GA group, indicating that BPPD plays an important role in upregulating the expression of osteogenic markers. Interestingly, in comparison with the GA and GA/BPPDM groups, the GA/BPPDM+NIR group showed the strongest improvement in inducing osteogenic marker expression, consistent with the ALP and ARS evaluation; this result demonstrated that the BPPD and NIR‐triggered DFO and PO_4_^3−^ release had a synergistic effect on promoting the expression of both early and late osteogenic markers. It is acknowledged that BP not only can be used as a photothermal agent, but also has biological activity and participates in the mineralization process, which induces osteogenesis by activating multiple signaling pathways, including the Wnt/β‐catenin and Ras/MAPK signaling pathways.^[^ ^14^ ^]^ On the other hand, the DFO released from the on‐demand delivery hydrogel also contributed to the enhancement of osteogenic activity.^[^ ^52^ ^]^ Similar trends were also detected for osteogenic marker protein expression in the immunofluorescence staining assay. As displayed in Figure 7I and Figure S17G (Supporting Information), the immunofluorescence staining assay revealed that both MC3T3‐E1 cells and BMSCs in the GA/BPPDM group secreted more osteogenic (Runx2 and OPN) proteins than those in the GA group, which also revealed the pro‐osteogenic activity of BPPD. More importantly, among all the groups, the GA/BPPDM hydrogel with heat stimulation showed the highest expression of Runx2 and OPN upon NIR irradiation (Figure S18, Supporting Information), which represented an excellent improvement in bone formation. Overall, these data demonstrated that the GA/BPPDM+NIR group had a prominent stimulatory effect on the osteogenic differentiation of both MC3T3‐E1 cells and BMSCs and probably promoted bone healing by upregulating the gene expression of ALP, Runx2, Col‐1, OPN, and OCN.

### 2.7 In Vitro Evaluation of the Effect of Immunomodulation on Angiogenesis and Osteogenesis

**Figure 8:**
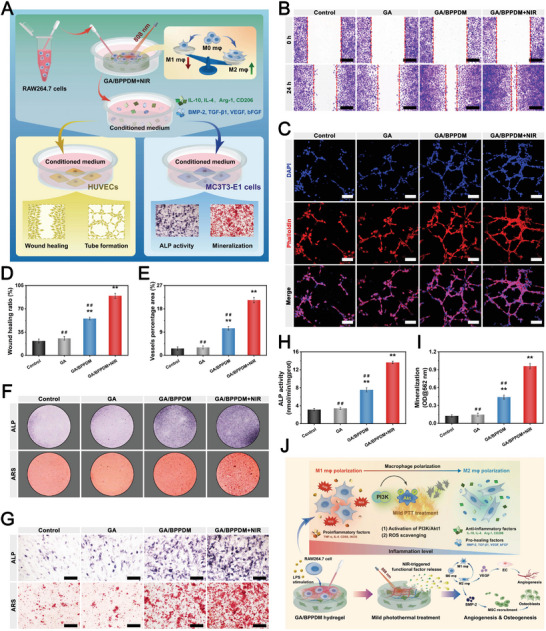
A) Schematic diagram of exploring the effect of conditioned medium collected from activated macrophages on angiogenesis and osteogenesis. B) Crystal violet staining and D) quantitative analysis of the wound healing assay. C) CLSM images of the tube formation assay and E) quantitative analysis of vessel percentage area. F,G) Macroscopic and microscopic images of ALP staining and ARS staining of MC3T3‐E1 cells. Quantitative analysis of H) ALP activity and I) mineralization level of MC3T3‐E1 cells. J) Schematic illustration of the potential mechanism of anti‐inflammation and M2‐type macrophage polarization and the subsequent guiding effect on vascularization and osteogenic differentiation by the GA/BPPDM hydrogel under mild NIR irradiation. Scale bar: 200 µm (B,C,G). Data are presented as the mean ± SD (*n* = 3). *p < 0.05 and **p < 0.01 indicate significant differences compared with the control group. ^#^ *p* < 0.05 and ^# #^ *p* < 0.01 indicate significant differences compared with the GA/BPPDM+NIR group.

During the process of bone regeneration, activated M2 macrophages participate in the clearance of debris, suppression of inflammation, and regulation of angiogenesis and osteogenesis.^[^ ^56^ ^]^ As mentioned in the previous sections, the photoactivated GA/BPPDM hydrogel platform with excellent anti‐inflammatory and immunomodulatory properties could not only promote M2 macrophage polarization but also produce a conducive immune microenvironment through the secretion of various cytokines, such as IL‐4, IL‐10, BMP‐2, TGF‐β1, VEGF, and bFGF. Consequently, in this section, we used conditioned medium to assess the effect of macrophage phenotype reprogramming on angiogenic and osteogenic responses, as shown in the schematic illustration (**Figure** **8**A). The conditioned medium derived from macrophages treated with GA, GA/BPPDM and GA/BPPDM plus NIR irradiation was collected and used for subsequent in vitro wound healing, tube formation, ALP activity and ARS staining assays. In terms of angiogenic activity, the GA/BPPDM+NIR group elicited a robust ability to promote HUVEC migration and tube formation, as indicated by the enhanced wound healing rate and vessel percentage area (Figure 8B–E). Meanwhile, the ALP activity and calcium mineral deposition of MC3T3‐E1 cells in the GA/BPPDM+NIR group were significantly higher than those in the other groups (Figure 8F–I), which was mainly related to the anti‐inflammatory and therapeutic cytokines secreted by M2 macrophages. Another reason behind the angiogenic and osteogenic activities may also be attributed to the presence of BP and DFO.

Taken together, these in vitro results suggested that the GA/BPPDM hydrogel system could not only directly promote osteogenic and angiogenic differentiation, thus accelerating bone regeneration but also indirectly increase the secretion of various pro‐healing cytokines in the microenvironment through immunomodulation, thereby accelerating osteogenesis and angiogenesis. This was consistent with previous studies showing that M2 macrophages played a positive role in recruiting mesenchymal progenitor cells, boosting angiogenesis and osteogenic differentiation by secreting various factors such as BMP‐2 and VEGF.^[^ ^57^ ^]^ As illustrated in Figure 8J, we elucidated the underlying mechanism of GA/BPPDM hydrogel‐mediated anti‐inflammation, macrophage phenotype reprogramming and immunomodulatory function. Under the action of BPPD and mild PTT treatment, the GA/BPPDM hydrogel resulted in inhibited oxidative stress and diminished secretion of proinflammatory cytokines (CD86, IL‐6, TNF‐α, and iNOS) by activating the PI3K/Akt1 signaling pathway. As reported, the upregulation of the PI3K/Akt1 signaling pathway has been identified to be associated with the activation of M2 macrophages.^[^ ^58^ ^]^ In addition, ROS was one of the factors that could regulate the transformation of M1/M2 macrophages, while reducing local levels of ROS benefits the transformation of macrophages from the M1 phenotype to the M2 phenotype, leading to the secretion of a diverse array of anti‐inflammatory and pro‐healing signals for accelerated tissue regeneration,^[^ ^27^ ^]^ which is consistent with our in vitro immunomodulation results. Consequently, the NIR‐irradiated GA/BPPDM hydrogel system with favorable immunomodulatory and antioxidant activity could orchestrate M2 macrophage polarization and promote the production of anti‐inflammatory, angiogenic and osteogenic cytokines, recreating a favorable regenerative microenvironment for vascularization and osteogenesis. The results from both direct and indirect evaluations suggested that the GA/BPPDM hydrogel system not only had good biocompatibility, pro‐angiogenic and pro‐osteogenic activities, and ROS scavenging ability but also induced anti‐inflammatory M2‐type macrophage polarization to remodel the damaged microenvironment into a pro‐healing microenvironment for enhanced angiogenesis and osteogenesis.

### 2.8 In Vivo Immunomodulatory Properties, Angiogenesis, and Bone Regeneration Capabilities in SD Rat Skull Defect Models

**Figure 9:**
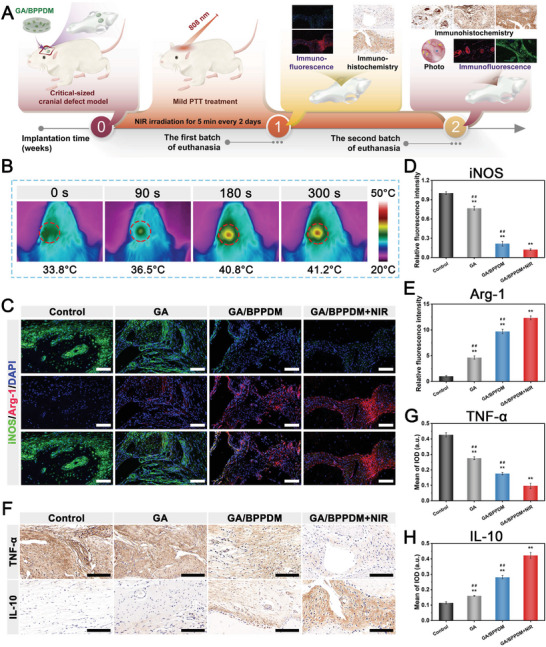
A) Schematic diagram for analysis of the early immune response, neovascularization, and stem cell recruitment. B) Real‐time infrared thermal images of rats implanted with GA/BPPDM hydrogel under NIR irradiation for 5 min (1 W cm^−2^, 808 nm). C) Immunofluorescence staining and D,E) quantitative analysis of iNOS and Arg‐1. F) Immunohistochemical staining and G,H) quantitative analysis of TNF‐α and IL‐10. Scale bar: 100 µm (C,F). Data are presented as the mean ± SD (*n* = 3). *p < 0.05 and **p < 0.01 indicate significant differences compared with the control group. ^#^ *p* < 0.05 and ^# #^ *p* < 0.01 indicate significant differences compared with the GA/BPPDM+NIR group.

Based on the results described above, our prepared GA/BPPDM hydrogels possessed favorable osteogenic and angiogenic capabilities as well as efficient immunomodulatory performance, showing great potential in accelerating bone tissue repair. However, whether these beneficial effects can be achieved in vivo remains unclear. In this study, a critical‐sized skull defect model (Φ = 5 mm) in rats was constructed to further evaluate the influence of GA/BPPDM with NIR irradiation on regulating the regenerative microenvironment and accelerating bone healing. A schematic diagram of our in vivo study is illustrated in **Figure** **9**A. The GA/BPPDM hydrogel was implanted into the defect sites as a photoactivated material followed by NIR laser irradiation to maintain a local temperature of 41 ± 1 °C (Figure 9B), which is suitable for tissue regeneration without additional photothermal injury.^[^ ^35^ ^]^ During the early stage of bone defects and material implantation, the inflammatory response initially occurs and further determines the fate of bone healing by inducing immune cells to migrate toward wound sites to accommodate the immune microenvironment.^[^ ^59^ ^]^ Therefore, at 7 days after the operation, the immunomodulatory ability of GA/BPPDM+NIR on the bone defect microenvironment was investigated by immunofluorescence staining. iNOS and Arg‐1 were chosen as typical surface markers for M1 or M2 macrophages, respectively. As shown in Figure 9C, a substantial increase of iNOS‐positive macrophages was detected in the control group after bone defect modeling, indicating a serious inflammatory state. Simultaneously, the three experimental groups not only significantly decreased the percentage of iNOS‐positive macrophages but also increased the percentage of CD206‐positive macrophages infiltrated in the defect sites, especially in the GA/BPPDM+NIR group, which exhibited the highest M2 expression, implying a weakened inflammatory state. These data suggested that the GA/BPPDM+NIR group could convert proinflammatory M1 macrophages to anti‐inflammatory M2 macrophages, thus efficiently reducing the local inflammatory response during bone healing. The fluorescence intensity also showed that GA/BPPDM+NIR exhibited higher CD206^+^ expression and lower iNOS^+^ expression (Figure 9D,E), which was consistent with the decreased M1 phenotype markers (CD86, IL‐6, TNF‐α, and iNOS) and improved levels of M2 phenotype markers (IL‐4, IL‐10, Arg‐1, and CD206) measured by qRT‐PCR assay (Figure S19, Supporting Information). To further validate the regulatory effect of the implanted hydrogels on macrophage polarization in the defect area, the representative proinflammatory cytokine TNF‐α and anti‐inflammatory cytokine IL‐10 were investigated on day 7. As presented in Figure 9F, the defect site in the control group induced the highest expression of TNF‐α, suggesting a serious inflammatory response. As expected, downregulated proinflammatory cytokine (TNF‐α) and upregulated anti‐inflammatory cytokine (IL‐10) were observed in both the GA/BPPDM and GA/BPPDM+NIR groups in comparison with the control and GA groups. And this was particularly obvious in the GA/BPPDM+NIR group, which exhibited significantly higher expression of IL‐10 (Figure 9G,H), indicating an excellent anti‐inflammatory effect. The role of PDA and mild heat stimulation in anti‐inflammation and macrophage polarization has been widely studied.^[^ ^60^ ^]^ Researchers found that NIR‐derived mild thermal stimulation at 41 ± 1 °C was able to protect cells from ROS‐induced oxidative damage through inhibition of the NF‐κB signaling pathway, thus promoting tissue regeneration under chronic inflammatory conditions.^[^ ^47^ ^]^ Besides, the combination of PDA and mild PTT triggered by NIR laser irradiation could significantly reprogram macrophages from the M1 phenotype to the M2 phenotype, thereby suppressing inflammatory responses and producing a pro‐regenerative microenvironment for bone healing. Combined with the previous results both in vitro and in vivo, it was demonstrated that the GA/BPPDM hydrogel was beneficial for polarizing macrophages toward the M2 phenotype, and mild heat stimulation further ameliorated the inflammatory environment, thus creating a conducive microenvironment for subsequent tissue regeneration.

**Figure 10:**
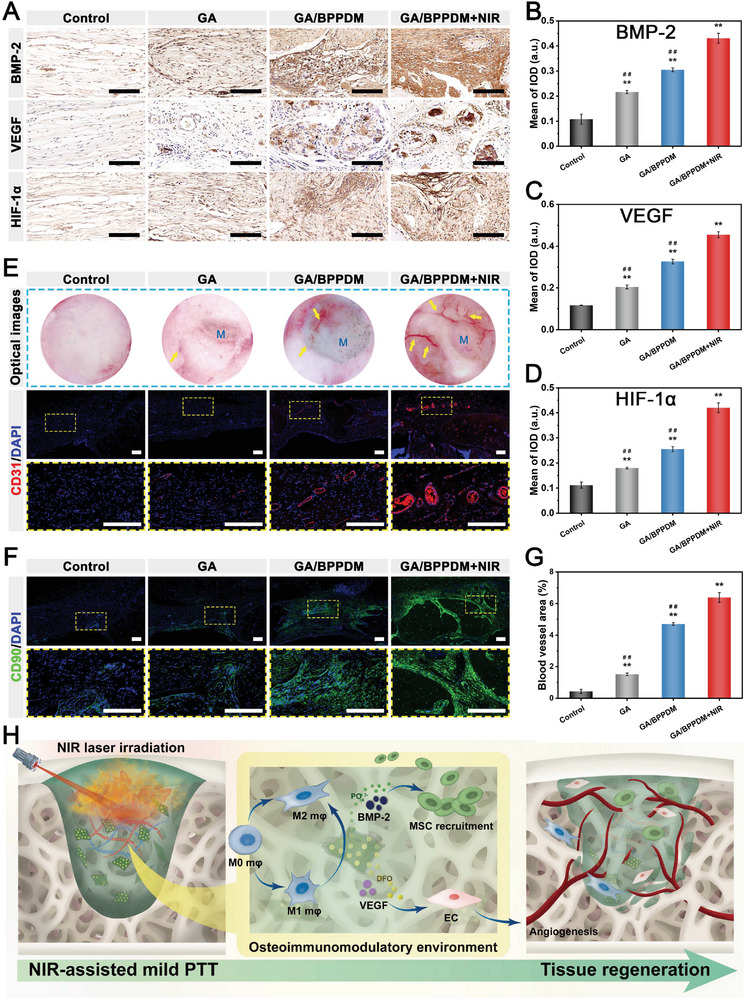
A) Immunohistochemical staining and B–D) quantitative analysis of BMP‐2, VEGF, and HIF‐1α. E) Photographs and immunofluorescence staining of CD31 in the defect region. F) Immunofluorescence staining of CD90 in the defect region. G) Quantitative analysis of CD31‐positive blood vessels. H) Schematic diagram of early immunomodulation and subsequent promotion effect on neovascularization and endogenous stem cell recruitment. Scale bar: 100 µm (A), 200 µm (E,F). Data are presented as the mean ± SD (*n* = 3). *p < 0.05 and **p < 0.01 indicate significant differences compared with the control group. ^#^ *p* < 0.05 and ^# #^ *p* < 0.01 indicate significant differences compared with the GA/BPPDM+NIR group.

There is substantial evidence that the presence of M2 macrophages can induce high expression of BMP‐2 and VEGF in the regenerative microenvironment, acting as potent inducers of osteogenesis and angiogenesis to stabilize bone and blood vessel formation.^[^ ^57^ ^]^ The early pro‐osteogenic and pro‐angiogenic capacities of different hydrogels were further verified by immunohistochemical staining, including BMP‐2, VEGF, and HIF‐1α (**Figure** **10**A). Owing to the synergistic interaction of the GA/BPPDM hydrogel system and mild heat stimulation, the GA/BPPDM+NIR group effectively enhanced the secretion of pro‐regenerative factors in the microenvironment of bone defects, with significant BMP‐2 and VEGF marker expression observed at week 2 post‐treatment (Figure 10B,C). Similarly, HIF‐1α, as an upstream regulator that can activate the transcription of VEGF, was also remarkably upregulated in the GA/BPPDM+NIR group (Figure 10D). Multiple studies have identified that BMP‐2 and VEGF are the most effective cytokines in promoting osteogenesis and vascular regeneration, respectively, while BMP‐2 can indirectly promote angiogenesis by stimulating VEGF.^[^ ^61^ ^]^ Furthermore, as an important regulator of angiogenesis, HIF‐1α can stimulate the growth of new blood vessels and provoke transcription of its downstream molecule VEGF.^[^ ^62^ ^]^ The quantitative analysis results further showed that the expression of BMP‐2, VEGF, and HIF‐1α was significantly higher in the GA/BPPDM+NIR group than in the other groups (Figure 10B–D), which could beneficially achieve enhanced cellular responses for rapid neovascularization, promoted recruitment of MSCs to the defect site, and enhanced ECM biosynthesis during the proliferation phase.

In the process of bone development and bone remodeling, enhanced neovascularization and endogenous stem cell recruitment were demonstrated to accelerate new bone formation.^[^ ^31^ ^]^ After 2 weeks of bone defect modeling, there was degradation of the residual hydrogels to various degrees in the defect area, which integrated well with the surrounding tissues without obvious displacement. Moreover, all hydrogel‐treated groups presented newly formed blood vessels (yellow arrows) within the defect area, in remarkable contrast to the limited vascularization observed in the control group (Figure 10E). Notably, the GA group showed a small amount of vascular network, while the GA/BPPDM and GA/BPPDM+NIR groups displayed a more obvious vascular structure, indicating abundant blood vessel infiltration, which is also consistent with the results of in vitro tube formation and subcutaneous embedding experiments. Especially in the GA/BPPDM+NIR group, abundant microvessel ingrowth was detected throughout the whole defect area (Figure 10E), resulting in a complete vascular network, which was mainly due to the secreted VEGF and HIF‐1α as well as the presence of an M2 macrophage‐enriched pro‐healing microenvironment. Meanwhile, by detecting the expression of CD31 (a specific marker of vascular endothelial cells) and CD90 (a specific surface marker of stem cells), we further analyzed angiogenesis as well as the recruitment and migration of endogenous stem cells during the repair of the defect site. The results of immunofluorescence staining showed that almost no signals for stem cell accumulation (CD90^+^, green) and only a small amount of host vascular endothelial cells (CD31^+^, red) with sporadic distribution were detected in the control group at 2 weeks (Figure 10E,F). Conversely, both GA/BPPDM and GA/BPPDM+NIR promoted endogenous stem cell recruitment and newly formed blood vessel infiltration into the defect area, especially for GA/BPPDM+NIR, which showed a more developed ring‐shaped vascular system and higher enrichment of CD90^+^ MSCs. Quantitative results for CD31 and CD90 expression also confirmed that GA/BPPDM plus mild heat stimulation could facilitate the ingrowth of host blood vessels as well as the recruitment of endogenous stem cells (Figure 10G; Figure S20, Supporting Information), which were crucial for the osseointegration of host bone tissue and implanted material in the early stage of bone repair. Herein, Figure 10H illustrates the osteoimmunomodulatory mechanism of the GA/BPPDM therapeutic platform. Benefiting from the combination of GA/BPPDM and mild PTT treatment, GA/BPPDM+NIR could recreate a favorable osteoimmunomodulatory microenvironment (anti‐inflammatory and M2‐polarizing) and drive the production of osteogenic/angiogenic factors (BMP‐2 and VEGF) to induce endogenous MSC recruitment and blood vessel formation, thereby initiating robust osteogenesis and angiogenesis. Collectively, owing to bioactive components and functional properties, the GA/BPPDM photothermal hydrogel could effectively transform the tissue microenvironment from inflammatory to reparative by polarizing macrophages recruited at the defect to the M2 type and promoting the secretion of anti‐inflammatory and pro‐healing cytokines, thereby achieving augmented bone regeneration.

**Figure 11:**
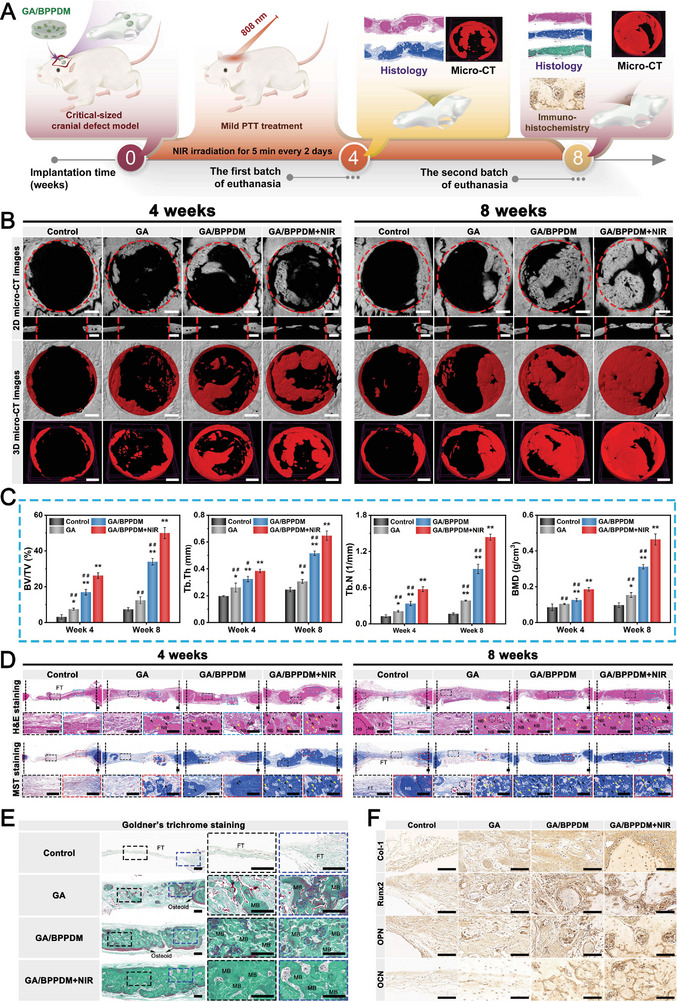
A) Schematic diagram for the analysis of bone regeneration. B) 2D‐ and 3D‐reconstructed micro‐CT images, including transverse and coronal sections, in the defect sites at 4 and 8 weeks after implantation. C) Quantitative analysis of micro‐CT results, including BV/TV, Tb.Th, Tb.N, and BMD. D,E) Histological analysis, including H&E staining, MST staining, and Goldner's trichrome staining, of regenerated bone tissue in the defect sites (FT: fibrous tissue, NB: newly formed bone tissue, HB: host bone, black arrow: newly formed bone lacunae, yellow arrow: newly formed central canal, black circle: residual hydrogel, MB: mineralized/mature bone (green), blue circle: immature bone (osteoid, red)). F) Immunohistochemical staining of Col‐1, Runx2, OPN, and OCN in the defect area. Scale bar: 1 mm (B), 200 µm (D,E), 100 µm (F). Data are presented as the mean ± SD (*n* = 3). *p < 0.05 and **p < 0.01 indicate significant differences compared with the control group. ^#^ *p* < 0.05 and ^# #^ *p* < 0.01 indicate significant differences compared with the GA/BPPDM+NIR group.

Then, the bone regeneration performance was analyzed using micro‐CT, histological staining, and immunohistochemical staining. A schematic diagram of the bone regeneration capacity experiment is presented in **Figure** **11**A. From 2D‐ and 3D‐reconstructed micro‐CT images, only a small amount of new bone distributed around the rim of the defect areas could be found in the control group at both 4 and 8 weeks. As expected, a critical‐sized skull defect model was successfully established in the present study. In sharp contrast, a significantly greater amount of new bone ingrowth was detected in the GA/BPPDM and GA/BPPDM+NIR groups at 4 weeks, which subsequently formed a more complete bone structure at 8 weeks (Figure 11B). Especially in the GA/BPPDM+NIR group, the density of newly formed bone tissue was dramatically increased, with the defect site almost completely healed at 8 weeks, while only sporadic new bone tissue appeared in the GA group. The examination of the coronal view also reflected the same result, showing that a complete bone bridge connecting the defects could be detected in the GA/BPPDM+NIR group. However, visible and obvious defects were still present in the GA and control groups without such apparent bone formation at the defect edge due to the limited self‐healing capacity. These results further highlighted the synergistic role of the GA/BPPDM hydrogel combined with mild photothermal treatment in promoting osteogenesis and tissue regeneration in vivo. It is worth noting that the micro‐CT results in the GA group were better than those in the control group in vivo, which is mainly attributed to the fact that GA with biomimetic bone ECM structure could also promote bone regeneration despite without any modification, consistent with previous studies.^[^ ^4^ ^]^

Accordingly, quantitative analysis of the micro‐CT examination is shown in Figure 11C, revealing the formation of new bone in the defect area. The analysis of bone regeneration‐related parameters, including the bone tissue volume/total tissue volume (BV/TV), trabecular thickness (Tb.Th), trabecular number (Tb.N), and bone mineral density (BMD), was consistent with the micro‐CT results. The BV/TV of the GA/BPPDM+NIR group reached 50.01 ± 3.04% at 8 weeks, which was significantly higher than that of the GA/BPPDM group (34.01 ± 1.77%), GA group (12.49 ± 1.97%), and control group (7.35 ± 0.97%), indicating that the presence of BPPD and mild PTT can promote new bone formation in vivo. Furthermore, the critical index for evaluating bone regeneration efficacy, including Tb.Th (0.64 0.04 mm), Tb.N (1.44 ± 0.0.5 mm^−1^), and BMD (0.46 ± 0.03 g cm^−3^) in the GA/BPPDM+NIR group showed the highest value, followed by the GA/BPPDM and GA groups at 8 weeks, similar to the results of the BV/TV analysis, further verifying a fulfilled effect in the promotion of bone mass. Owing to the excellent NIR/pH‐dual‐responsive properties, the photothermal effect can increase the release of DFO rapidly to promote vascularization in the early stage of bone healing and sustainably release PO_4_^3−^ from the GA/BPPDM hydrogels to induce bone regeneration through in situ biomineralization during the long‐term bone repair period. Additionally, previous studies have reported that the combination of on‐demand mild hyperthermia (≈45 °C) and BP‐incorporated scaffolds could synergistically promote bone regeneration due to the alteration of cytoskeleton and integrin signaling as well as the upregulation of heat shock protein.^[^ ^63^ ^]^ Our study further proved that NIR‐induced mild PTT combined with the dual‐sensitive drug/ion delivery GA/BPPDM hydrogel platform had a significant promotion effect on bone healing in vivo. Taken together, relying on the sequential modulation of the immune microenvironment, revascularization, and osteogenesis, the NIR‐irradiated GA/BPPDM hydrogel platform can effectively promote bone regeneration in vivo, and the newly formed bone possesses excellent mineral density and intact bone structure.

**Figure 12:**
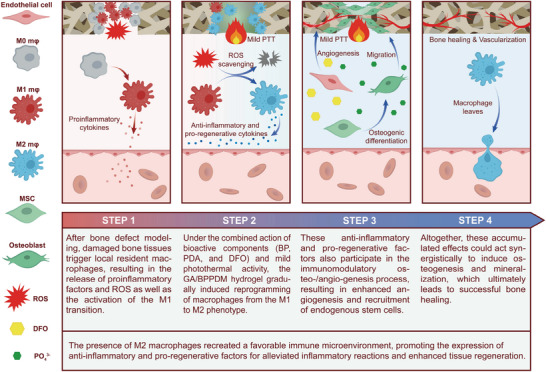
Schematic diagram of the possible mechanism by which the smart‐responsive multifunctional GA/BPPDM therapeutic system promotes bone regeneration.

Subsequently, the histopathological structures of the regenerated new bone in the defect sites were verified by H&E, Masson's trichrome (MST), and Goldner's trichrome staining. As seen from the histological images, the defect areas of the control group showed predominantly fibrous tissue formation bridging the edge of the host bone at 4 and 8 weeks (Figure 11D), implying poor bone regeneration potential, consistent with the results of the micro‐CT analysis. In contrast, there were numerous continuously regenerated lamellar bone with newborn bone lacunae and central canals observed in the GA/BPPDM and GA/BPPDM+NIR groups, which exhibited a relatively complete laminar structure. In particular, the defect areas of the GA/BPPDM+NIR group were almost completely occupied by dense and mature newly formed bone tissue, and the thickness was similar to that of the original bone at 8 weeks after implantation. MST staining further demonstrated that only a few collagen fibers were sparsely deposited within the defect area of the control group, while with the treatment of GA/BPPDM plus mild PTT, abundant collagen with dense and continuous structures was observed in the GA/BPPDM+NIR group, demonstrating the formation of mature lamellar bone. It is worth mentioning that the residual hydrogel material was almost replaced with fibrous tissue and surrounded by internal newly formed bone islands due to the ECM‐mimicking 3D microenvironment and proper degradation rate, which allowed space for new bone formation and ingrowth. Another possible reason is the creation of favorable regenerative microenvironment by the combined effect of GA/BPPDM and mild PTT (Figure 10H), which is conducive to tissue ingrowth and microcirculation, consequently accelerating the catabolism of the implanted hydrogel.^[^ ^64^ ^]^ These results indicated that the GA/BPPDM hydrogel combined with mild heat stimulation could promote cell ingrowth and facilitate material‐neo‐bone tissue integration, thus leading to quicker and better bone healing with bone‐like structures. Goldner's trichrome staining was further performed to evaluate the degree of bone maturation at 8 weeks after implantation (Figure 11E). Compared with the control and GA groups, a significantly greater amount of mature lamellar bone tissues (green) together with less immature bone (osteoid, orange/red) was observed in the GA/BPPDM group, indicating that the GA/BPPDM hydrogels facilitated bone mineralization and remodeling. Furthermore, after treatment with mild PTT, considerable, green‐stained mature bone tissues were observed to grow from the edges of defective regions toward the central areas, which meant that the new bone was gradually calcified and matured. This phenomenon fully demonstrated that new bone tissue tended to regenerate from the margin toward the center of defects, benefiting from the guiding and promoting role of the GA/BPPDM hydrogel and NIR‐triggered mild PTT on osteoprogenitor cell recruitment, migration, differentiation, and biomineralization. Hydrogel biodegradation in vivo was also assessed by H&E staining at 8 weeks after implantation. It could be found that the residual hydrogel material (yellow arrows) gradually degraded, and some tissue cells were observed within the interior of the hydrogels, indicating good compatibility and long‐term biodegradability in vivo (Figure S21, Supporting Information), which is beneficial for cell infiltration in the bone defect areas. Furthermore, the residual GA/BPPDM hydrogel was surrounded by internal newly formed bone islands, indicating the ability of the GA/BPPDM scaffold plus mild heat stimulation to facilitate bone tissue ingrowth and promote material‐tissue integration. To verify the ability of the smart‐responsive photothermal hydrogel platform to promote osteogenesis and biomineralization during bone repair, immunohistochemical staining of Col‐1, Runx2, OPN, and OCN was conducted at 8 weeks post‐implantation. As displayed in Figure 11F, the expression of these osteogenic markers in both the GA/BPPDM and GA/BPPDM+NIR groups was significantly higher than that in the control and GA groups. In particular, the GA/BPPDM+NIR group exhibited the strongest positive staining of Col‐1, Runx2, OPN, and OCN proteins, followed by those in the GA/BPPDM and GA groups (Figure S22, Supporting Information), indicating that the mild photothermal effect of GA/BPPDM could facilitate matrix maturation and mineralization during bone regeneration and remodeling. As mentioned above, GA/BPPDM plus NIR treatment can improve the local microenvironment and realize stimuli‐responsive release of DFO and PO_4_^3−^, resulting in the continuous secretion of these ECM proteins in the defect sites, thus accelerating tissue repair and regeneration. In addition, compared with the control group, no obvious pathological changes in major organs, including the heart, lung, liver, spleen, and kidney, were observed for all experimental groups, suggesting good biosafety of the hydrogels in vivo (Figure S23, Supporting Information). In this work, the superior performance of the GA/BPPDM hydrogel platform in the bone augmentation effect is mainly attributed to its multifunctional therapeutic features (**Figure** **12**). i) Owing to the combined action of BP, PDA, and DFO, GA/BPPDM could continuously maintain a conducive pro‐regenerative microenvironment in the repair process because of its superior osteogenic and angiogenic activities, which are vital for bone defect repair. ii) Under mild NIR irradiation, GA/BPPDM could modulate the polarization of macrophages toward the M2 phenotype and promote the production of anti‐inflammatory, angiogenic and osteogenic cytokines, thereby enhancing neovascularization and endogenous stem cell recruitment, which played a key role in the early stage of the healing process. Meanwhile, BPPD, as an important component of GA/BPPDM, could endow the hydrogel with mild photothermal activity and pH‐responsive and NIR laser‐triggered drug/ion release behavior for efficient bone regeneration. iii) Under the combined long‐term effect of physical (mild hyperthermia) and chemical (drug/ion delivery) interventions, the smart‐responsive multifunctional therapeutic system achieved improved regenerative microenvironment and efficient bone regeneration. Overall, the photoactivated GA/BPPDM hydrogel was a promising biomaterial scaffold that could augment the repair and regeneration of large bone defects, as indicated by accumulated M2‐type macrophages, reduced inflammation, promoted angiogenesis, and enhanced bone matrix deposition.

## 3 Conclusion

In summary, in this study, a smart‐responsive multifunctional hydrogel platform combined with NIR‐triggered mild PTT was developed for spatiotemporally manipulating macrophage polarization and vascular development for rapid bone regeneration. Owing to the photothermal conversion performance of BP and PDA as well as the pH sensitivity of the PDA layer, the as‐prepared dual‐drug delivery hydrogel system possessed spatial distributions of angiogenic (DFO) and osteogenic (PO_4_^3−^) factors under on‐demand NIR irradiation, thus achieving a synergistic therapeutic effect of immunomodulation, angiogenesis, and osteogenesis. The in vitro evaluation and in vivo subcutaneous implantation experiments demonstrated that combined with mild PTT under appropriate NIR irradiation, the GA/BPPDM therapeutic system had excellent biocompatibility, pro‐osteogenic and pro‐angiogenic capacities, as well as outstanding ROS‐scavenging capacities and immunomodulatory functions. The in vivo experiments further confirmed that NIR‐assisted GA/BPPDM could promote multiple regenerative processes in the healing process of a critical‐sized bone defect model in rats, including M2 polarization of macrophages, neovascularization, osteogenesis, and tissue remodeling. In conclusion, by combining physical (mild PTT treatment) and chemical (drug/ion delivery) interventions, the hydrogel ensured a rapid transformation from M1 to M2 macrophages at the early stage of inflammation and the secretion of pro‐osteogenic and pro‐angiogenic factors, which efficiently stimulated vascular tissue growth and endogenous stem cell recruitment and ultimately accelerated the bone repair process. This study offers an amazing strategy for bone regeneration through the combination of smart responsive design and bioactive factors, and multifunctional hydrogels provide broad implications for bone defect repair applications.

## Conflict of Interest

The authors declare no conflict of interest.

## Author Contributions

M.W., H.L., D.L., and Y.Z. contributed equally to this work. M.W. performed conceptualization, methodology, investigation, wrote the original draft, and project administration. H.L., D.L., Y.Z., performed conceptualization, methodology, investigation, and wrote the original draft. P.W., Z.C., F.C., Y.C., performed investigation. Z.D. performed conceptualization, methodology, wrote the original draft, and supervised. L.C. performed conceptualization, methodology, wrote the original draft, supervised, and funding acquisition.

## Supporting information

Supporting Information

## Data Availability

The data that support the findings of this study are available in the supplementary material of this article.
