# Supplementary material for: Smart‐Responsive Multifunctional Therapeutic System for Improved Regenerative Microenvironment and Accelerated Bone Regeneration via Mild Photothermal Therapy
Source: Adv Sci (Weinh). 2023 Nov 7;11(2):2304641. doi: 10.1002/advs.202304641 (PMC10787108; doi:10.1002/advs.202304641)
Supplement: Supplementary file 1 — Supporting Information [file ADVS-11-2304641-s001.pdf]

ADVANCED SCIENCE
Open Access

Supporting Information

for *Adv. Sci.*, DOI 10.1002/advs.202304641

Smart-Responsive Multifunctional Therapeutic System for Improved Regenerative Microenvironment and Accelerated Bone Regeneration via Mild Photothermal Therapy

Minhao Wu, Huifan Liu, Dan Li, Yufan Zhu, Ping Wu, Zhe Chen, Feixiang Chen, Yun Chen, Zhouming Deng and Lin Cai**

Supporting Information

Smart-responsive multifunctional therapeutic system for improved regenerative microenvironment and accelerated bone regeneration via mild photothermal therapy

Minhao Wu[#a], Huifan Liu[#b], Dan Li[#c], Yufan Zhu[#a], Ping Wu[d], Zhe Chen[a], Feixiang Chen[e], Yun Chen[e], Zhouming Deng[*a], Lin Cai[*a]

[a] Department of Spine Surgery and Musculoskeletal Tumor, Zhongnan Hospital of Wuhan University, 168 Donghu Street, Wuchang District, Wuhan 430071 Hubei, People's Republic of China

[b] Department of Anesthesiology, Research Centre of Anesthesiology and Critical Care Medicine, Zhongnan Hospital of Wuhan University, Wuhan, Hubei, China

[c] Department of Neonatology, Xianning Central hospital, School of Basic Medical Sciences, Xianning Medical College, Hubei University of Science and Technology, Xianning, Hubei, 437100, P.R. China

[d] Research Units of Clinical Translation of Cell Growth Factors and Diseases Research, Chinese Academy of Medical Science, 325000, Zhejiang, China

[e] Department of Biomedical Engineering and Hubei Province Key Laboratory of Allergy and Immune Related Disease, TaiKang Medical School (School of Basic Medicine Sciences), Wuhan University, Wuhan 430071, China

[#] These authors contributed equally to this work
* Correspondence should be addressed to:

Dr. Lin Cai. Email: orthopedics@whu.edu.cn
Department of Spine Surgery and Musculoskeletal Tumor, Zhongnan Hospital of Wuhan University, 168 Donghu Street, Wuchang District, Wuhan 430071 Hubei, People's Republic of China

Dr. Zhouming Deng. Email: dengzhouming@whu.edu.cn
Department of Spine Surgery and Musculoskeletal Tumor, Zhongnan Hospital of Wuhan University, 168 Donghu Street, Wuchang District, Wuhan 430071 Hubei, People's Republic of China

Supplementary experimental section

1. Materials and methods

1.1. Materials

BP crystals were obtained from Nanjing XFNANO Materials Tech Co., Ltd (Nanjing, China). DFO was purchased from Med Chem Express (Monmouth Junction, NJ, USA). Gelatin, sodium alginate, methacrylic anhydride, N-methyl pyrrolidone (NMP), lithium phenyl (2,4,6-trimethylbenzoyl) phosphinate (LAP), dopamine hydrochloride (DA), lipopolysaccharide (LPS), and tris (hydroxymethyl) aminomethane (Tris) were purchased from Sigma-Aldrich Trading Co., Ltd. (Shanghai, China). Fetal bovine serum (FBS), Dulbecco's modified Eagle's medium (DMEM), alpha-modified Eagle's medium (α-MEM), phosphate-buffered saline (PBS), trypsin-EDTA, and penicillin/streptomycin (P/S) were obtained from Gibco Life Technologies Co. (Grand Island, USA) for cell culture in vitro. The MC3T3-E1 cell line, HUVEC line, and RAW264.7 cell line were supplied by the Institute of Biochemistry and Cell Biology of the Chinese Academy of Sciences (Shanghai, China). Cell counting kit-8 (CCK-8) was acquired from Dojindo Laboratories (Kumamoto, Japan). The live/dead cell staining kit was purchased from BestBio Biotechnologies (Shanghai, China). Triton X-100 (Sigma-Aldrich), DAPI (Sigma-Aldrich), and TRITC-labeled phalloidin (Invitrogen) were used for cell staining. TRIzol RNA extract kit, radioimmunoprecipitation assay (RIPA) lysis buffer, 5-bromo-4-chloro-3-indolyl phosphate/nitro blue tetrazolium (BCIP/NBT) Alkaline Phosphatase (ALP) Color Development Kit, 2′,7′-dichlorofluorescein diacetate (DCFH-DA) probe, and bicinchoninic acid (BCA) protein assay kit were purchased from Beyotime Biotechnology Co., Ltd. (Jiangsu, China). The ALP assay kit was provided by Jiancheng Biotech Institute (Nanjing, China). All chemicals were used without further purification. The water applied in all experiments was purified by a Milli-Q cycle purification system (Millipore, USA).

1.2. Fabrication and characterization of the BPPD nanomaterials

A process diagram of the fabrication of BPPD is shown in **Scheme 1A**. First, pristine BP nanosheets were prepared using a facile liquid exfoliation method according to the previous literature[1]. Briefly, 50 mg of BP crystal was dispersed and sonicated in an ice bath at a power of 300 W for 12 h in 50 mL NMP solution. The non-exfoliated BP was removed by centrifugation at 4000 rpm for 15 min, and the BP nanosheets in the supernatant were collected. The stand-by BP nanosheets were re-dispersed in deionized (DI) water after spinning at 12000 rpm for 15 min to remove NMP. Then, the collected BP nanosheets were thoroughly dried in a vacuum oven at 37°C and stored for further PDA modification and DFO loading.

Subsequently, the PDA layer was coated onto BP nanosheets through oxidative self-polymerization under alkaline conditions following a previously reported method[2]. Briefly, 50 mg of BP nanosheets was suspended in a Tris-HCl solution (pH = 8.5) containing DA (2 mg/mL, 100 mL). The mixture was stirred in the dark for 12 h to ensure PDA layer formation. After centrifuging for 5 min at 5000 rpm, the obtained BP@PDA (10 mg) nanosheets were incubated with 10 mL DFO aqueous solution (1 mg/mL) and stirred for 12 h. The resulting solution was freeze-dried to obtain the BPPD nanocomposite for further use. The morphologies of the synthesized BPPD nanomaterials were observed using transmission electron microscopy (TEM, JEM2100, Hitachi, Tokyo, Japan) and atomic force microscopy (AFM, SPA400 Seiko, Japan). The surface charge of the BPPD nanomaterials was measured by using a Zetasizer Nano-ZS PN3702 system (Malvern Instruments, Worcestershire, England). The chemical composition of the BPPD nanomaterials was characterized using a Fourier transform infrared spectrometer (FTIR, TNZ1-5700, Nicolet, USA).

1.3. Preparation of GA/BPPD nanocomposite hydrogels

GelMA was prepared according to a previously reported protocol[3]. Briefly, 1 g of gelatin powder was dissolved in PBS (pH = 7.4) with 300 rpm magnetic stirring for 6 h. Methacrylic anhydride (0.6 g/mL) was added to the gelatin solution, and the reaction was processed for 3 h at 50°C under constant stirring. Finally, the obtained GelMA reaction was dialyzed against DI water using a 12000-14000 Da membrane and purified at 40°C for 5 days. Finally, the white porous foam product was obtained by freeze-drying and stored at -80°C. Alg-MA was prepared following the same process as mentioned above. The synthesis of GelMA and Alg-MA was confirmed by using proton-1 nuclear magnetic resonance (^{1}H-NMR, AV500 MHz, Bruker, Switzerland) and Fourier transform infrared spectroscopy (FTIR, Nicolet iS50, Thermo Fisher Scientific, USA).

For the preparation of nanocomposite hydrogels, GelMA (7% w/v) and Alg-MA (3% w/v) foams were dissolved in DI water (10% w/v) at 50°C and stirred for 1 h to obtain a clarified solution. Thereafter, a certain amount of BPPD and LAP (0.3% w/v) was added into the former solution and vigorously stirred for 2 h protected from light to obtain a homogeneous prepolymer solution. The final polymer solution was placed into specific molds and photo-crosslinked with UV light (405 nm, 5 W) for 3 min. In this work, a series of BPPD-incorporated hydrogels (GA/BPPD) were named as GA, GA/BPPDL, GA/BPPDM, and GA/BPPDH hydrogels (**Table S1**), according to the different percentages of BPPD nanocomposites from low to high (0 wt%, 0.2 wt%, 0.5 wt%, and 1 wt%).

1.4. Characterization of the hydrogels

After gelation was completed, the gross morphologies of the prepared GA/BPPD hydrogels were observed using a digital camera. The microscopic architecture and surface topography of the freeze-dried hydrogels were characterized by scanning electron microscopy (SEM, VEGA3, TESCAN, Czech Republic) at a voltage of 20 kV. The pore size of the freeze-dried hydrogel was calculated using ImageJ software. After lyophilization, the hydrogels were scanned by micro-computed tomography (micro-CT, SkyScan 1276, Bruker, Germany) and reconstructed by CTvox software (Bruker, Germany) according to our established protocol[4]. The interconnected porosity of the hydrogels was analyzed by CTAn software (Bruker, USA) based on micro-CT data, as described previously[5]. The wettability of different hydrogels was investigated using a contact angle meter (SL200B, Solon Technology Science, Shanghai, China) at room temperature. For the compressive test, the hydrogels were prepared in the form of cylinders with 10 mm in diameter and 10 mm in height. The mechanical performance was conducted at room temperature on a universal testing machine (CMT6503, Shenzhen SANS Test Machine, China) with a compression rate of 1 mm/min. The maximum strain of the compressive test was fixed at 50%. The rheological properties of different samples were tested by a rheometer (Kinexus, Malvern) with a temperature control system, as previously reported[6]. The rheological properties (storage modulus G' and loss modulus G'') of different samples were measured by a dynamic thermomechanical analyzer (DMA, Thermo, USA). The strain and frequency values were selected in the linear region. The swelling ratio of the hydrogel samples was determined by measuring the weight of the freeze-dried hydrogel samples before (D0) and after (D1) immersion in the PBS solution at predetermined time points. The swelling ratio = D1/D0 × 100%. The degradation behavior of the hydrogel samples was calculated as follows. The initial weight of the freeze-dried hydrogel samples (W0) was recorded; then, the hydrogels were immersed in hybrid degradation solutions containing 100 mg/mL lysozyme in a shaking incubator at 37°C and 120 rpm. At scheduled time points, the dry weight of the remaining hydrogel samples was weighed (W1). The remaining mass ratio = W1/W0 × 100%.

1.4.1. Photothermal performance of GA/BPPD hydrogels

For the photothermal conversion test, the hydrogel samples were added into 24-well plates containing PBS and then irradiated by an 808 nm NIR laser (KS-810F-8000, Kai Site Electronic Technology, China) at a power density of 1 W/cm^2. During irradiation, the temperature and corresponding thermal images of different samples were recorded by a FLIR ONE thermal imager (FLIR Systems, Inc., Wilsonville, OR) at different time intervals. Besides, the photothermal stability of GA/BPPD hydrogels was evaluated with 808 nm laser irradiation at a 1 W/cm^2 power density by turning the NIR laser on and off.

1.4.2. In vitro evaluation of pH-responsive and NIR-triggered release kinetics of GA/BPPD hydrogels

To investigate the release behavior of DFO, a standard curve of DFO was first drawn as previously described. The amount of DFO in the supernatant was then examined via a UV-Vis spectrophotometer (Perkin Elmer, USA) according to the standard calibration curve. The loading efficiency was calculated using the following equation: drug loading efficiency (%) = weight of the loaded DFO/weight of the final product. To evaluate the NIR- and pH-triggered drug release kinetics, the GA/BPPD hydrogel was immersed in 10 mL of PBS with different pH values (6.5 and 7.4) at 37°C under constant shaking for 4 weeks. At the predetermined time point, 5 mL of the supernatant was collected and replaced with the same volume of fresh PBS solution. The amount of DFO in the supernatant was determined according to the corresponding calibration curve of the standard DFO solution. The NIR-triggered release behavior was also determined under the same conditions at pH values = 6.5 and 7.4 and irradiation with an 808 nm NIR laser (5 min, 1 W/cm^2). Meanwhile, the concentration of PO_4^{3-} released from the GA/BPPD composite hydrogels was detected by immersion in Tris-HCl (pH = 7.4) for 28 days. The leaching solutions of the samples were collected and measured with ion chromatography (ICS-2500, Dionex, USA).

1.5. In vitro evaluation of cytocompatibility

In this work, both MC3T3-E1 preosteoblastic cells and HUVECs were employed for in vitro evaluations of cytocompatibility, as they are the major and important cell source for bone regeneration. Before cell seeding, all prepared hydrogels were immersed in 75% (v/v) ethanol solution for 24 h and sterilized with UV for 30 min followed by rinsing with PBS buffer. The cytocompatibility of the hydrogels was assessed by detecting the viability of both MC3T3-E1 preosteoblastic cells and HUVECs. In brief, MC3T3-E1 cells or HUVECs were co-cultured with the hydrogels at a density of 1 × 10^5 cells/mL. Pure GA hydrogel was used as the control group. The cell/hydrogel composites were maintained in DMEM supplemented with 10% FBS and 1% (v/v) P/S at 37°C in a 5% CO_2 humidified atmosphere. At predetermined time points, cell proliferation was assayed by using the CCK-8 assay according to the manufacturer's instructions. The optical density (OD) value at 450 nm was measured with a microplate reader (Multiskanfc, Thermo Scientific). After co-culturing for 3 days, live/dead staining was carried out to evaluate the cell viability of the hydrogels following the manufacturer's guidelines. Cells were treated with 1 mM calcein-AM/2 mM EthD-1 working solution for 15 min in the dark. Confocal laser scanning microscopy (CLSM; TCS SP8, Leica, Germany) was used to acquire the images. Live and dead cells were labeled in green and red, respectively. And then the cell density was calculated by ImageJ software. To observe the cell morphology of MC3T3-E1 cells or

HUVECs cultured on the hydrogels for 3 days, the cell cytoskeletons were stained with TRITC-labeled phalloidin for 1 h after fixation with 4% paraformaldehyde, and the nuclei were counterstained with DAPI for 5 min. Finally, the morphology and number of cells on the hydrogel were observed by CLSM. F-actin and nuclei were labeled in red and blue, respectively. The cell spreading area of cells on the different samples was quantified by ImageJ software.

1.6. In vitro evaluation of osteogenic potential

To detect the effect of NIR-triggered drug release on MC3T3-E1 cell migration, a co-culture Transwell assay was conducted, as previously described[7]. Briefly, the cells were placed in the upper chamber of the Transwell at a density of 1 × 10^4 cells per well, while the hydrogels were immersed in the basement of the Transwell with or without NIR irradiation (1 W/cm^2) for 5 min every 24 h. The peak irradiation temperature was maintained at 41 ± 1°C. After 12 h of incubation, the migrated cells in the lower chamber were stained with 0.1% crystal violet, rinsed with PBS, and then photographed by optical microscopy.

To investigate the effect of NIR-triggered drug release on the osteogenic differentiation of MC3T3-E1 cells, a Transwell co-culture model was applied as previously described[8]. Briefly, MC3T3-E1 cells at a concentration of 2 × 10^4 cells/well were seeded into the lower chamber of a 24-well Transwell plate for adhesion, and after 24 h, 100 μL of various hydrogels was placed in the upper chamber of the plate. The osteogenic induction medium was exchanged every three days. The hydrogels were placed in the upper chamber and divided into different groups according to the received treatments as follows: GA hydrogels, GA/BPPD hydrogels, and GA/BPPD hydrogels with 808 nm NIR laser irradiation (5 min, 1 W/cm^2). MC3T3-E1 cells were incubated in osteogenic induction medium (DMEM complete medium supplemented with 10 mM β-glycerophosphate disodium salt, 0.05 mM ascorbic acid, and 10 nM dexamethasone) for osteogenic differentiation. The osteogenic induction medium was exchanged every three days. On days 7 and 14, the cells were stained with a BCIP/NBT ALP color development kit, and the ALP activity assay was performed by using a commercial ALP assay kit and a BCA protein assay kit following each manufacturer’s instructions. On days 14 and 21, Alizarin red S (ARS) staining was performed to detect mineralized matrix formation. To quantify calcium deposition, 10% cetylpyridinium chloride (Sigma-Aldrich) was added to the wells with shaking. After centrifugation for 15 min, the absorbance of the supernatant was measured at a wavelength of 562 nm with a UV-Vis spectrophotometer.

To detect the gene transcription and protein expression levels of typical osteogenic markers, including ALP, Col-1, Runx2, OPN, and OCN, real-time polymerase chain reaction (qRT-PCR) and Western blot assays were performed on day 7. The mRNA

levels were normalized to the housekeeping gene GAPDH, and the data were calculated using the $2^{-\Delta\Delta CT}$ method. The primer sequences used in this study are presented in **Table S2**. In addition, immunofluorescence staining analysis was performed to verify the expression of osteogenesis-related markers. Briefly, after co-culturing for 7 days, the cells were fixed with 4% paraformaldehyde for 30 min, followed by permeabilization in 0.5% Triton X-100. After blocking with 1% bovine serum albumin for 1 h, the samples were incubated with primary antibodies, including anti-Runx2 (Cell Signaling, D1L7F) and anti-OPN (ProteinTech, 22952-1-AP), at 4°C overnight, and then treated with the corresponding secondary antibodies for 1 h in the dark. After washing with PBS, the cells were counterstained with DAPI for 5 min. Finally, fluorescence images were observed with CLSM and analyzed by using ImageJ software.

Furthermore, bone marrow-derived mesenchymal stem cells (BMSCs) isolated from the femurs and tibiae marrow of Sprague-Dawley (SD) rats were also used for osteogenic activity evaluation in vitro[9]. BMSCs at passages 3-5 with good growth were cultured in osteogenic induction medium and tested for ALP activity, ASR staining, qRT-PCR and immunofluorescence staining assays as mentioned above.

1.7. In vitro evaluation of angiogenic activity

To investigate the effect of NIR-triggered drug release on angiogenesis in vitro, the migration ability of HUVECs was assessed by wound healing and Transwell assays, as previously described[10]. For the wound healing assay, HUVECs were cultured in a 6-well plate and incubated for 12 h to ensure cell adhesion. Afterwards, a linear scratch was created with a 10 μL pipette tip on the HUVEC monolayer, and the cells were rinsed with PBS to remove cellular debris. After incubation for 24 h, the cells were fixed with 4% paraformaldehyde, stained with 0.1% crystal violet, and then observed under an optical microscope. For the Transwell assay, HUVECs were seeded into the upper chamber in 24-well Transwell plates. After 24 h, the migration of HUVECs was photographed using an optical microscope and counted using ImageJ software. To detect the angiogenesis of HUVECs in vitro, a tube formation assay was performed according to the manufacturer’s protocol. Briefly, HUVECs were seeded onto Matrigel-coated 24-well plates and then incubated at 37°C to form a vascular-like network structure. After 8 h of incubation, TRITC-labeled phalloidin and DAPI were used to stain the cytoskeleton and nucleus, respectively, as mentioned above. The labeled cells were observed by CLSM, and the average tube formation parameter was quantified using AngioTool (National Cancer Institute, NIH). After HUVECs were co-cultured with the hydrogels for 7 days as mentioned above, qRT-PCR analysis was performed to evaluate genes (Ang-1, bFGF, eNOS, HIF-1α, and VEGF) related to angiogenesis. The relative gene expression level was normalized to the housekeeping gene GAPDH and calculated using the $2^{-\Delta\Delta CT}$

method. The primer sequences are shown in **Table S2**. For immunofluorescence staining, cells were washed and fixed in 4% paraformaldehyde for 30 min, permeabilized with 0.1% Triton and blocked with 1% bovine serum albumin for 30 min. Next, the samples were incubated with primary target antibodies against CD31 (ProteinTech, 11265-1-AP), VEGF (ProteinTech, 66828-1-Ig), and HIF-1α (20960-1-AP) at 4°C overnight. After incubation with the fluorescent-labeled secondary antibody, the samples were counterstained with DAPI for 5 min at room temperature and then observed with CLSM. Fluorescence intensity was determined via ImageJ software.

1.8. In vitro evaluation of immunomodulatory performance

To investigate the immunomodulatory effects of the hydrogels on macrophage responses in vitro, a 3D co-culture model was established for the culture of macrophage/hydrogel composites as previously described. To induce macrophages toward M1 phenotype polarization, LPS (100 ng/mL) was used to stimulate macrophages for 2 days to mimic the inflammatory environment. Next, RAW264.7 macrophages at a density of 2 × 10^5/mL were seeded onto the hydrogels and then subjected to periodical NIR irradiation (1 W/cm^2, 808 nm) for 5 min each day. The peak irradiation temperature was maintained at 41 ± 1°C. Cells cultured in dishes without hydrogel served as controls. To study the intracellular ROS-scavenging activity of hydrogels, cells were incubated with DCFH-DA (25 μM), followed by incubation for 20 min in the dark. Subsequently, DCFH-DA fluorescence in the cells was examined under a CLSM. Additionally, the intracellular ROS levels were assessed by flow cytometry (FC500, Beckman Coulter, Fullerton, CA, USA). For detection of the macrophage response, after incubation for 4 days, the macrophage/hydrogel composites were fixed with 4% paraformaldehyde for 30 min, permeabilized with 0.5% Triton X-100, and then blocked with 1% bovine serum albumin for 1 h. All the composites were then treated with rabbit polyclonal anti-F4/80 antibody (ProteinTech, 28463-1-AP) overnight at 4°C, followed by incubation with Cy3-conjugated goat anti-rabbit IgG (Abclone, AS007) for 1 h. Finally, the nuclei and cytoskeleton were counterstained with DAPI and FITC-labeled phalloidin and subjected to CLSM observation. Additionally, to observe cell infiltration, the cell nucleus was stained with DAPI when macrophages were cultured in different hydrogels for 4 days. Then, the infiltration of macrophages within the hydrogels was imaged using CLSM. For flow cytometry analysis, the cells were stained with C86 antibody (Biolegend, 105011) and CD206 antibody (Biolegend, 141703). All data were acquired on a flow cytometer (FC500, Beckman Coulter, Fullerton, CA, USA) and analyzed with FlowJo software (Tree Star). Immunofluorescence staining analysis was also performed to verify the expression of macrophage polarization-related markers. Briefly, the macrophage/hydrogel composites were fixed and penetrated following the same process as mentioned above after

incubation for 4 days. The samples were then incubated with primary antibodies against iNOS (1:200, ab210823, Abcam) and CD206 (1:200, PA5-101657, Invitrogen) at 4°C overnight, followed by further incubation with secondary antibodies for 30 min in the dark. After 5 min of nuclear staining with DAPI, the images were photographed by CLSM.

The expression levels of inflammation-related genes in macrophages co-cultured on different samples for 4 days were assessed by qRT-PCR, and GAPDH was used as an internal reference as mentioned above. For the Western blot assay, RAW264.7 macrophages after treatment were lysed, and the total protein levels were quantitatively analyzed using a BCA protein assay kit. Equal concentrations of protein in each sample were subjected to sodium dodecyl sulfate-polyacrylamide gel electrophoresis (SDS-PAGE), and then transferred onto polyvinylidene difluoride (PVDF) membranes. Subsequently, the membrane containing proteins was blocked for 1 h in 5% non-fat milk, then incubated with primary antibodies at 4°C overnight, followed by rinsing with TBST. After that, they were incubated with the corresponding secondary antibodies for 1 h at room temperature. Finally, the membranes containing protein bands were visualized by a Tanon-5200 chemiluminescent imaging system (Tanon, Shanghai, China). Furthermore, immune-related cytokines in the medium were measured by commercial ELISA kits (Meimian, Jiangsu, China) according to the manufacturer’s instructions.

To further investigate the influence of macrophage phenotype reprogramming on vascularization and osteogenesis induction, the conditioned medium of different groups was collected for subsequent study based on previously reported protocols[11]. After co-culturing for 4 days, the cell supernatants of each group were collected by centrifugation at 5000 rpm for 5 min and filtered through a 0.22 μm filter. The conditioned medium was composed of fresh DMEM and the above supernatant (1:1, v/v). For angiogenesis evaluation, HUVECs in the different conditioned medium were subjected to wound healing and tube formation assays with the same method mentioned above. For osteogenesis evaluation, MC3T3-E1 cells were incubated in osteogenic induction medium (conditioned medium supplemented with 10 mM β-glycerophosphate disodium salt, 0.05 mM ascorbic acid, and 10 nM dexamethasone). The osteogenic induction medium was changed every two days. At scheduled time points, the osteogenic potential of MC3T3-E1 cells was assessed in terms of ALP activity and ARS staining using the methods described above.

1.9. In vivo evaluation of immunomodulatory and angiogenic effects in a subcutaneous implantation model

The procedures of all animal studies in this study were approved by the Animal Ethical Committee of Wuhan University, and the methods in the current work were carried out in strict accordance with “Guiding Opinions on the Treatment of Animals (09/30/2006)”

published by the Ministry of Science and Technology of the People's Republic of China. Prior to the bone regeneration study, the ability of the multifunctional hydrogel platforms to modulate macrophage polarization and vascularization was investigated using a dorsal subcutaneous implantation model in rats as previously described[12]. Because the pure GelMA hydrogel had no obvious photothermal effect, three groups of GA hydrogel, GA/BPPDM, and GA/BPPDM+NIR irradiation were applied to the subcutaneous study. In detail, eighteen male SD rats (6-8 weeks old, 250 ± 15 g) in a specific pathogen-free (SPF) animal room were randomly divided into three groups (6 rats per group) according to the following implantation conditions: (1) GA group (implanted with GA hydrogel), (2) GA/BPPDM group (implanted with GA/BPPDM hydrogel), and (3) GA/BPPDM+NIR group (implanted with GA/BPPDM hydrogel and then irradiated with 808 nm NIR light for 5 min every 2 days). During the NIR irradiation process, the local temperature of the wound was maintained at 41 ± 1°C with the aid of an FLIR infrared thermal imager that recorded the temperature of the implanted site in real time. After 7 days of implantation, some fresh hydrogel samples were collected for ELISA according to the manufacturer’s recommendations. Inflammation-related cytokines, including IL-10 and TNF-α, were then measured. For histopathological analysis, some samples were fixed with 4% paraformaldehyde, dehydrated, embedded, and sectioned for hematoxylin and eosin (H&E) staining. To evaluate the macrophage response in vivo, immunohistochemical analyses of iNOS (a specific marker of M1 macrophages) and CD206 (a specific marker of M2 macrophages) were performed. Furthermore, immunohistochemical analyses of CD31 (expressed by endothelial cells) and α-smooth muscle actin (α-SMA) (expressed by vascular smooth muscle cells) were also conducted to evaluate angiogenic activity in vivo.

1.10. In vivo evaluation of the immune response, neovascularization, and bone formation in an SD rat skull defect model

A critical-sized skull defect model was established in male SD rats to evaluate the in vivo immune response, neovascularization, and bone regeneration capacities, which was approved by the Animal Care and Use Committee of Wuhan University. In brief, sixty SD rats (6-8 weeks old, 250 ± 15 g) were randomly assigned to four groups (15 rats per group): (1) control group (bone defects without implantation), (2) GA group (implanted with GA hydrogel), (3) GA/BPPDM group (implanted with GA/BPPDM hydrogel), and (4) GA/BPPDM+NIR group (implanted with GA/BPPDM hydrogel and then subjected to extra NIR irradiation for 5 min every 2 days). To explore the potential immunomodulatory and bone regeneration ability of mild PTT treatment in vivo, the area of rats treated with GA/BPPDM hydrogel received NIR laser radiation (808 nm, 1 W/cm^2) after implantation as mentioned above. Meanwhile, the local temperature was maintained at 41 ± 1°C for 5

min and monitored by an infrared thermal imager. After the rats were anesthetized by inhalation of isoflurane, the calvarial vault area of each rat was shaved and cleaned with iodine solution. After that, a middle-longitudinal incision was made over the calvarium, and two symmetrical circular defects (φ = 5 mm) were subsequently created by an electric drill. The sterilized hydrogels were carefully inserted, and the incision was closed layer-by-layer with 4-0 nylon sutures. All surgical operations were conducted by the same surgeons under sterile conditions. Considering that a high temperature may damage normal tissues near the defect, the PTT of the hydrogels was determined at a mild temperature (41 ± 1°C), which was controlled by cooling during irradiation. After 7 days of implantation, the harvested calvarial samples (n=6 for each group) were fixed with 4% paraformaldehyde and then decalcified using 10% EDTA solution at 37°C for 4 weeks. After paraffin embedding and sectioning, 5-μm-thick sections were prepared and processed for immunofluorescence and immunohistochemical staining according to previously described protocols[13]. To evaluate the macrophage response, immunofluorescence staining of iNOS (a specific marker of M1 macrophages) and Arg-1 (a specific marker of M2 macrophages) was performed. Simultaneously, some fresh samples were stored in liquid nitrogen to extract total RNA for inflammatory gene expression analyses. Immunohistochemical staining of TNF-α and IL-10 was further conducted to examine the early inflammatory status in the defect area after implantation. To assess the local neovascularization and recruitment of endogenous stem cells at 2 weeks after implantation, immunofluorescence staining of CD31 and CD90 was performed in the bone defect site. Additionally, immunohistochemical staining of BMP-2 (a potent marker for osteogenesis) and VEGF (a potent marker for angiogenesis) was carried out to evaluate the secretion of osteo-inductive and angio-inductive factors. Finally, the immunofluorescence and immunohistochemical staining results were analyzed by ImageJ software.

At week 4 and week 8 post-implantation, the harvested calvarial samples were scanned using a high-resolution micro-CT system (SkyScan 1276, Bruker, Germany). The scanning parameters were set at 55 kV, 185 μA, 0.2° rotation step, and filtration of 0.25 mm aluminum with a resolution of 6.5 μm per pixel. After scanning, 3D high-resolution reconstructed images were obtained and analyzed with NRecon, DataViewer, CTvox, and CTAn software. For histological and immunohistochemical analysis, serial 5 μm thick cross-sections of the samples were processed for H&E, Masson's trichrome (MST), and Goldner's trichrome staining according to the manufacturer's protocol. In addition, immunohistochemical staining of Col-1, Runx2, OPN, and OCN in the defect area was conducted according to a previously reported standard protocol. At 8 weeks after implantation, animal viscera, including the heart, liver, spleen, lung, and kidney in each group, were also collected for H&E staining to examine the long-term in vivo

biosafety of the implanted hydrogels.

1.11. Statistical analysis

All quantitative data in the study are presented as the mean ± standard deviation (mean ± SD) of three representative experiments. Data were analyzed using Origin 2018 software (Origin Lab Corporation, USA) by one-way ANOVA with Tukey's test. Data with abnormal distribution or heterogeneity of variance were analyzed by the Mann-Whitney U test and Kruskal-Wallis's nonparametric test. The values were considered significant at p* or $p^{\#}$, represented by a p value < 0.05, and highly significant at p** or $p^{\#\#}$, with a p value < 0.01.

Supplementary figures and tables

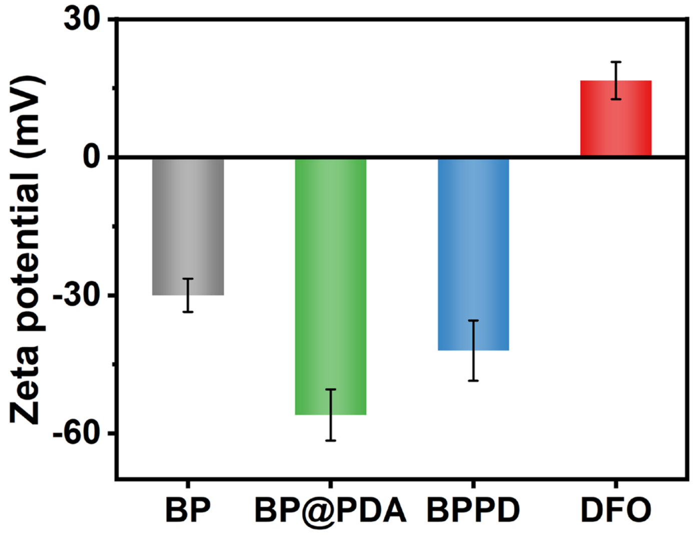

Fig. S1. Zeta potential of BP, BP@PDA, BPPD, and DFO. Data are presented as the mean ± SD (n = 3).

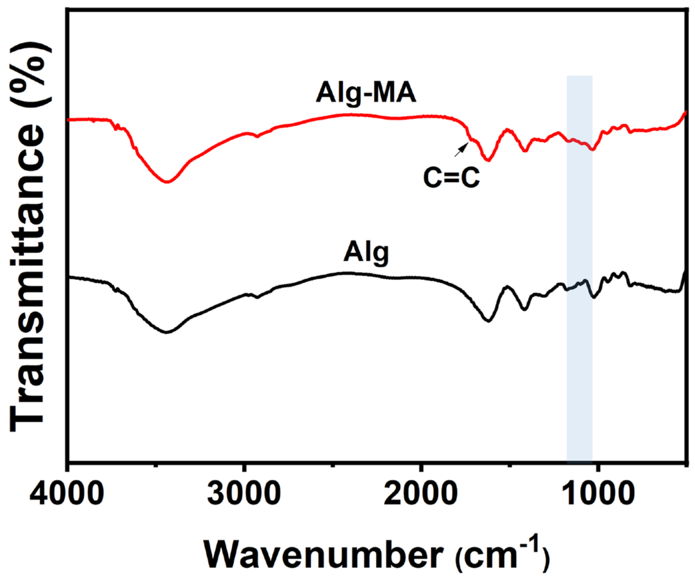

Fig. S2. FTIR spectra of Alg and Alg-MA.

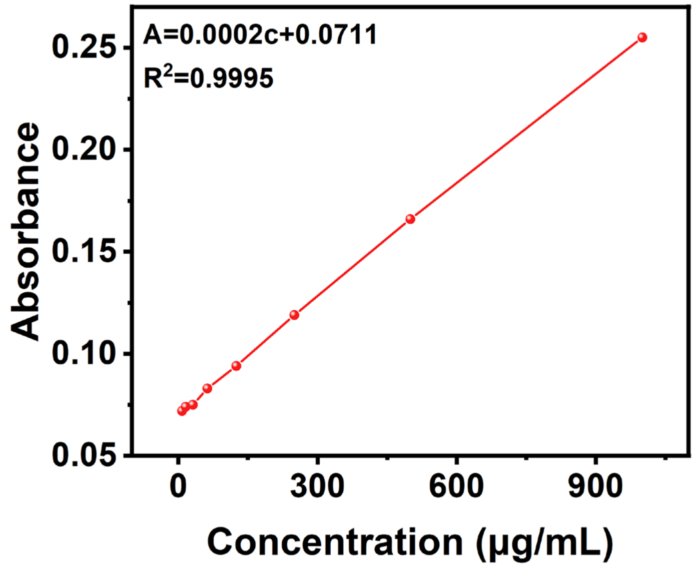

Fig. S3. Fitted standard curve of DFO.

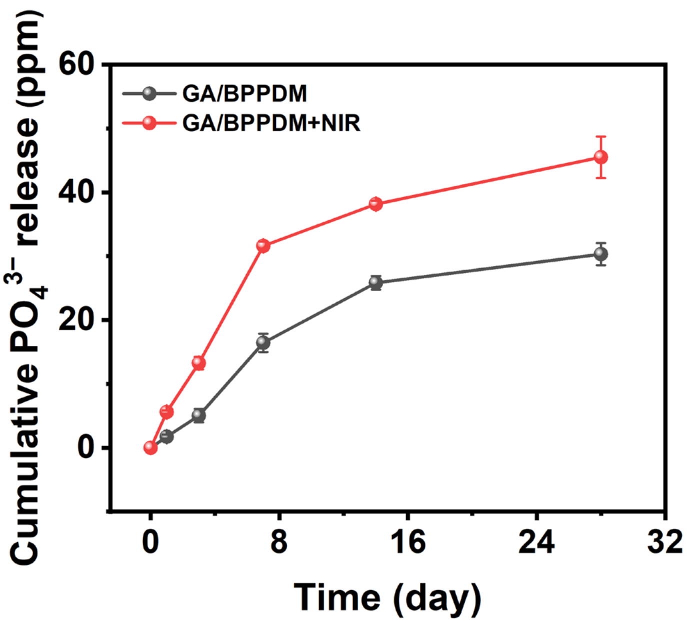

Fig. S4. Cumulative release curve of PO_4^{3-} from the GA/BPPDM hydrogel with or without NIR irradiation. Data are presented as the mean ± SD (n = 3).

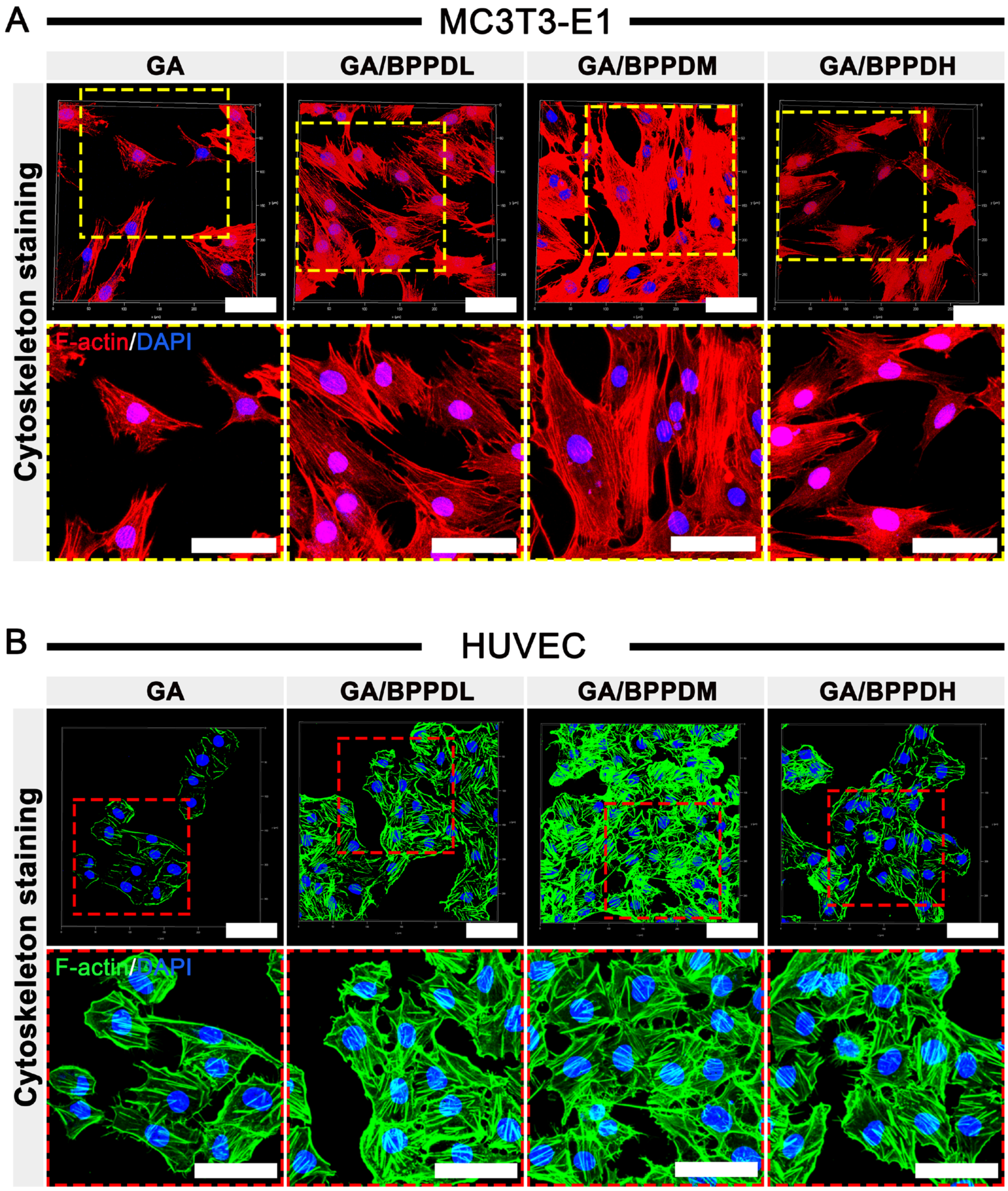

Fig. S5. Cytoskeleton fluorescence staining of F-actin and DAPI in **(A)** MC3T3-E1 cells and **(B)** HUVECs cultured on the hydrogels for 3 days. Scale bar: 75 μm (A, B).

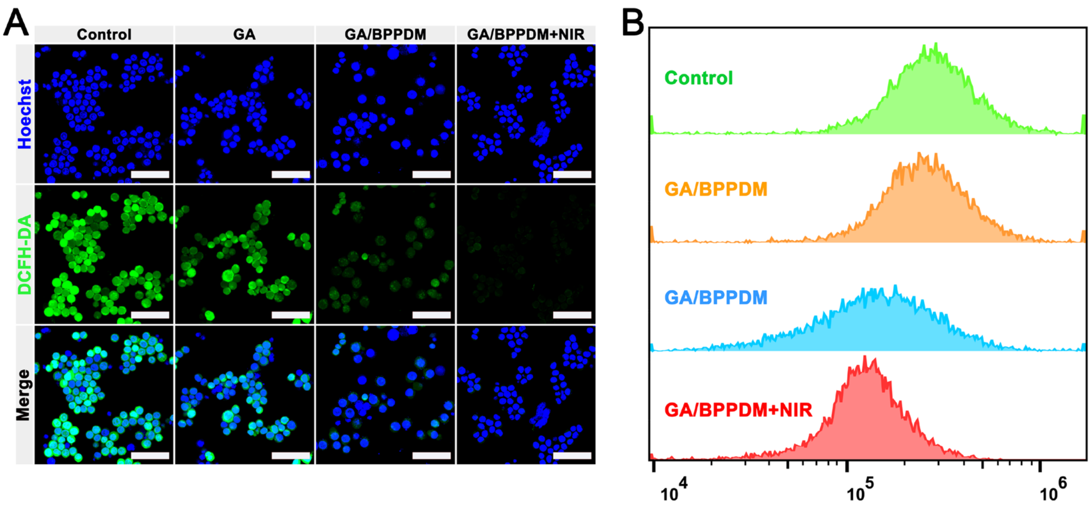

Fig. S6. **(A)** CLSM images and **(B)** flow cytometry analysis of ROS levels in RAW264.7 macrophages after treatment. Scale bar: 50 μm (A).

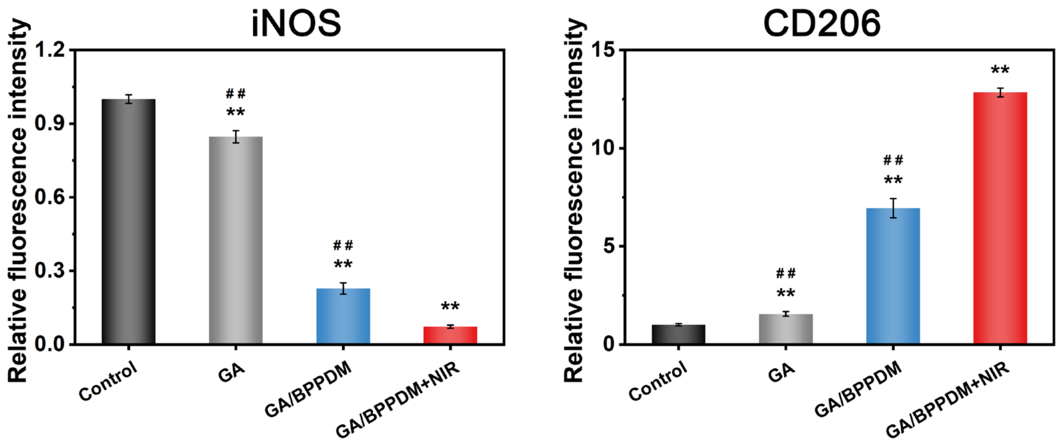

Fig. S7. Quantitative analysis of the fluorescence intensity of iNOS and CD206 in RAW264.7 macrophages. Data are presented as the mean ± SD (n = 3). *P < 0.05 and **P < 0.01 indicate significant differences compared with the control group. $^{\#}$P < 0.05 and $^{\#\#}$P < 0.01 indicate significant differences compared with the GA/BPPDM+NIR group.

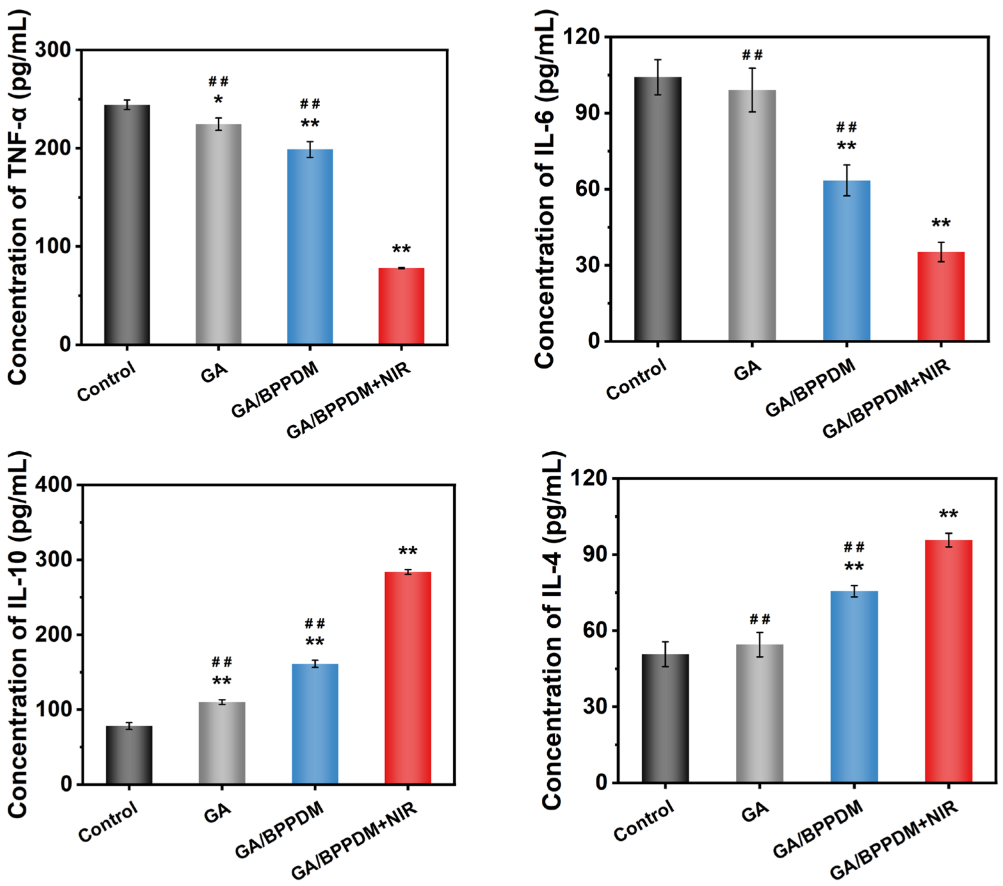

Fig. S8. ELISA analysis of M1 cytokines (TNF-α, IL-6) and M2 cytokines (IL-10, IL-4) in cell culture supernatants. Data are presented as the mean ± SD (n = 3). *P < 0.05 and **P < 0.01 indicate significant differences compared with the control group. $^{\#}$P < 0.05 and $^{\#\#}$P < 0.01 indicate significant differences compared with the GA/BPPDM+NIR group.

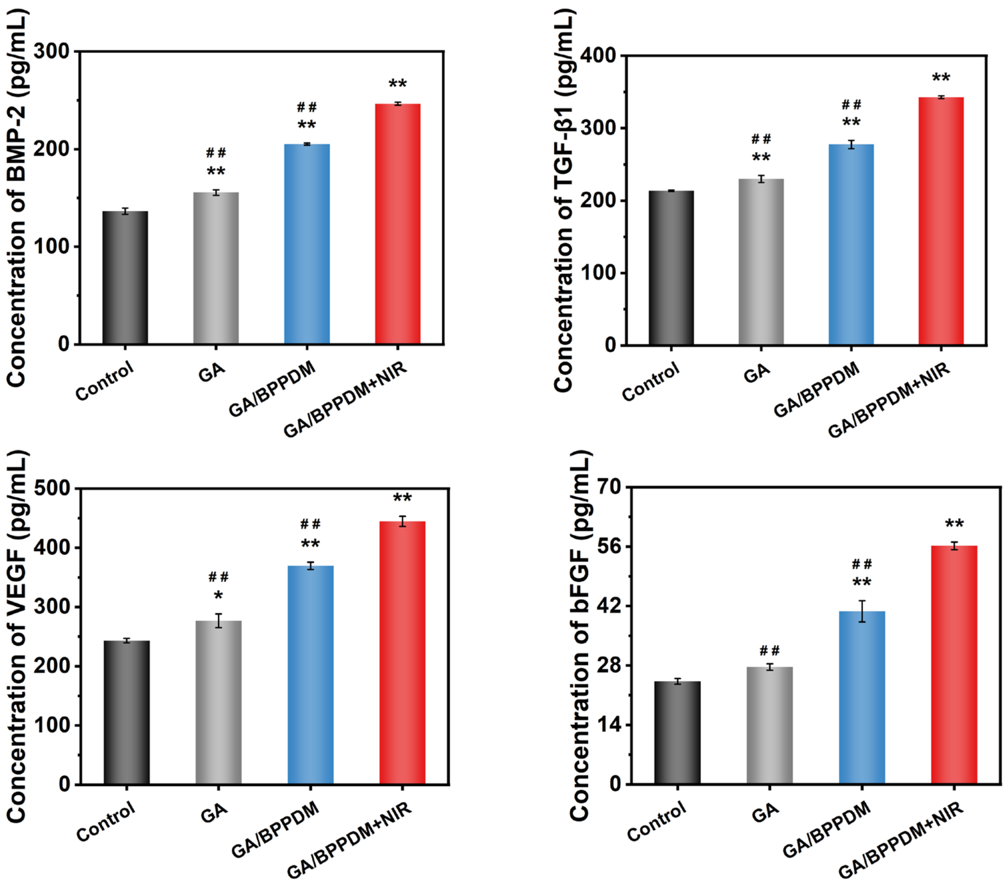

Fig. S9. ELISA analysis of pro-osteogenic cytokines (BMP-2, TGF-β1) and pro-angiogenic cytokines (VEGF, bFGF) in cell culture supernatants. Data are presented as the mean ± SD (n = 3). $^{*}P < 0.05$ and $^{**}P < 0.01$ indicate significant differences compared with the control group. $^{\#}P < 0.05$ and $^{\#\#}P < 0.01$ indicate significant differences compared with the GA/BPPDM+NIR group.

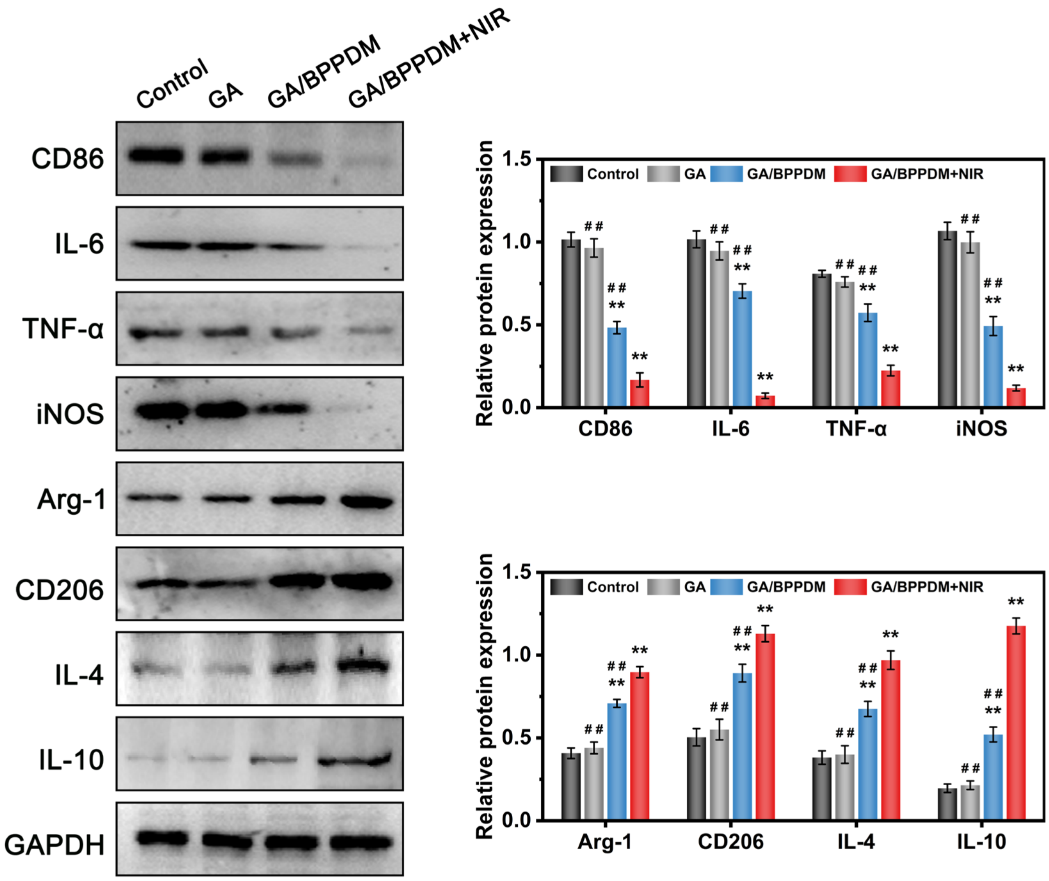

Fig. S10. Western blot analysis and protein expression of M1 (CD86, IL-6, TNF-α, and iNOS) and M2 (IL-4, IL-10, Arg-1, and CD206) macrophage markers. Data are presented as the mean ± SD (n = 3). *P < 0.05 and **P < 0.01 indicate significant differences compared with the control group. $^{\#}$P < 0.05 and $^{\#\#}$P < 0.01 indicate significant differences compared with the GA/BPPDM+NIR group.

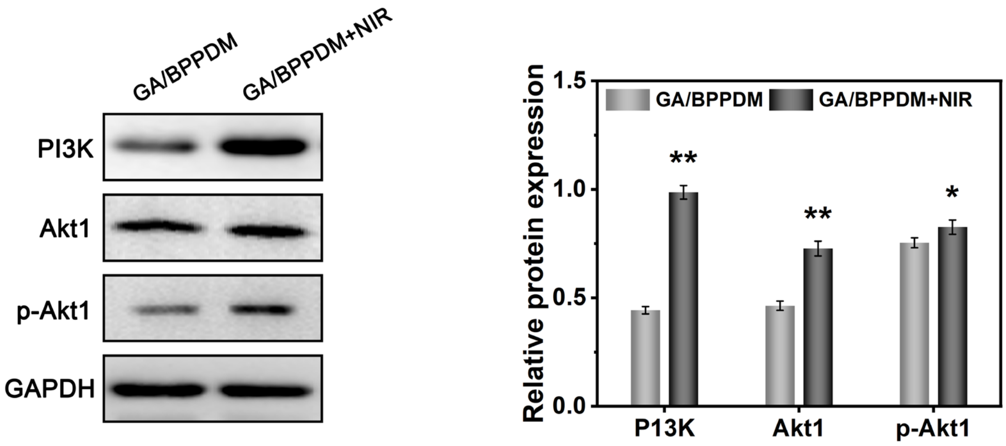

Fig. S11. Western blot analysis and protein expression of PI3K, Akt1, and p-Akt1 in RAW264.7 macrophages cultured on GA/BPPDM with or without mild PTT treatment for 4 days. Data are presented as the mean ± SD (n = 3). *P < 0.05 and **P < 0.01 indicate significant differences compared with the GA/BPPDM group.

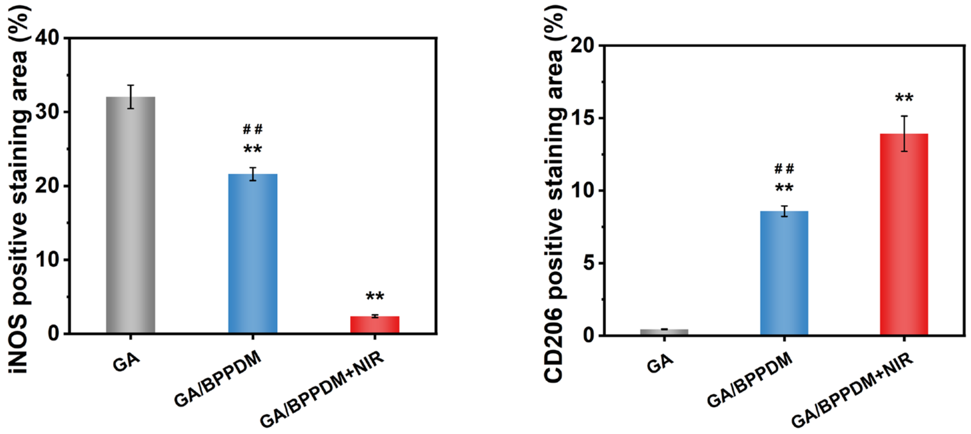

Fig. S12. Quantification of iNOS- and CD206-positive staining areas in different hydrogels on day 7 after implantation in the rat subcutaneous model. Data are presented as the mean ± SD (n = 3). *P < 0.05 and **P < 0.01 indicate significant differences compared with the GA group. $^{\#}$P < 0.05 and $^{\#\#}$P < 0.01 indicate significant differences compared with the GA/BPPDM+NIR group.

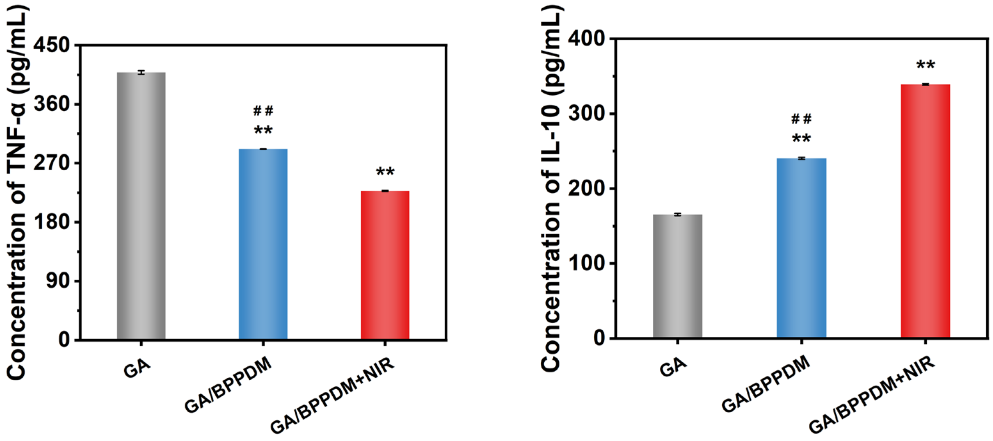

Fig. S13. ELISA analysis of TNF-α and IL-10 from the hydrogel sample lysates extracted on day 7 after implantation in the rat subcutaneous model. Data are presented as the mean ± SD (n = 3). *P < 0.05 and **P < 0.01 indicate significant differences compared with the GA group. $^{\#}$P < 0.05 and $^{\#\#}$P < 0.01 indicate significant differences compared with the GA/BPPDM+NIR group.

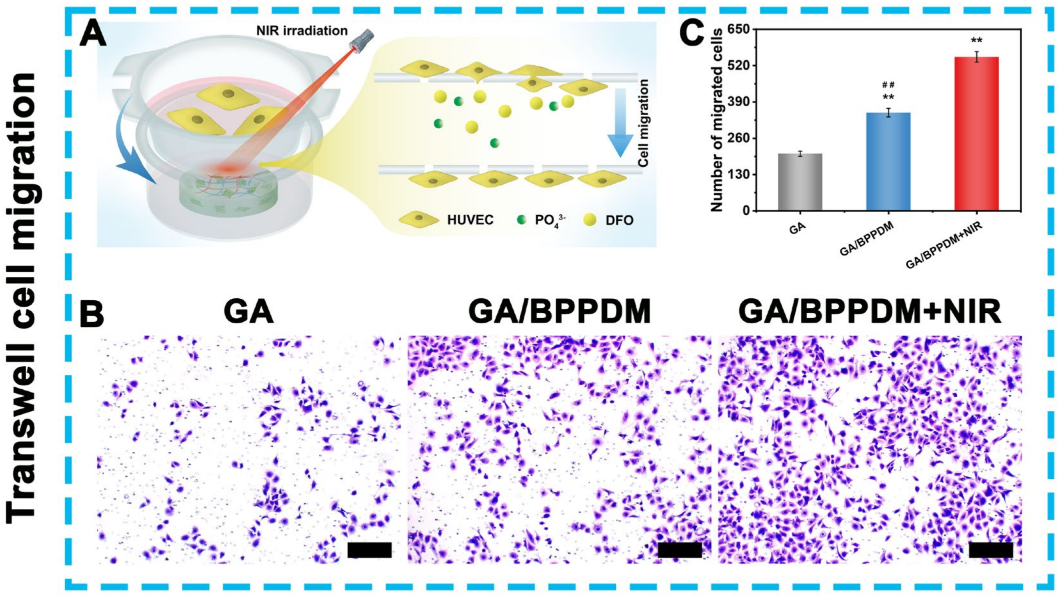

Fig. S14. (A) Schematic diagram of exploring the migration of HUVECs in a Transwell assay. **(B)** Crystal violet staining and **(C)** quantitative analysis of migrated HUVECs. Scale bar: 200 μm (B). Data are presented as the mean ± SD (n = 3). *P < 0.05 and **P < 0.01 indicate significant differences compared with the GA group. #P < 0.05 and # #P < 0.01 indicate significant differences compared with the GA/BPPDM+NIR group.

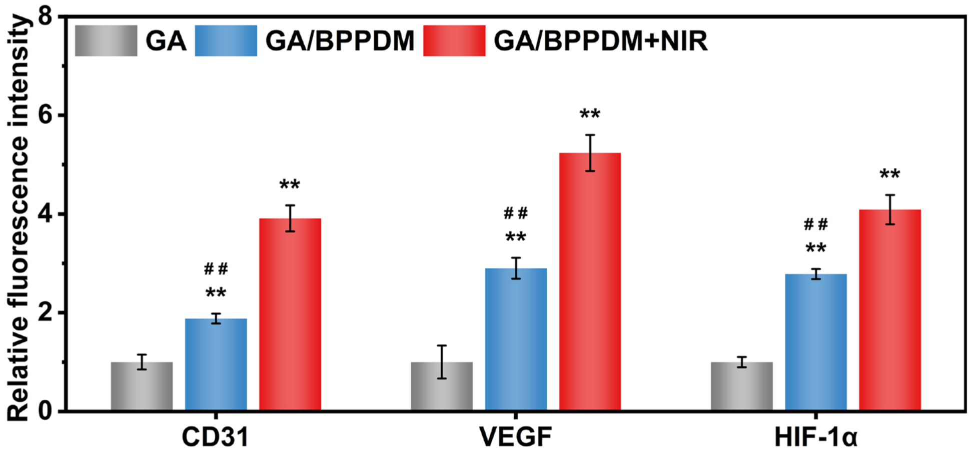

Fig. S15. Quantitative analysis of the fluorescence intensity of CD31, VEGF, and HIF-1α in HUVECs. Data are presented as the mean ± SD (n = 3). *P < 0.05 and **P < 0.01 indicate significant differences compared with the GA group. #P < 0.05 and # #P < 0.01 indicate significant differences compared with the GA/BPPDM+NIR group.

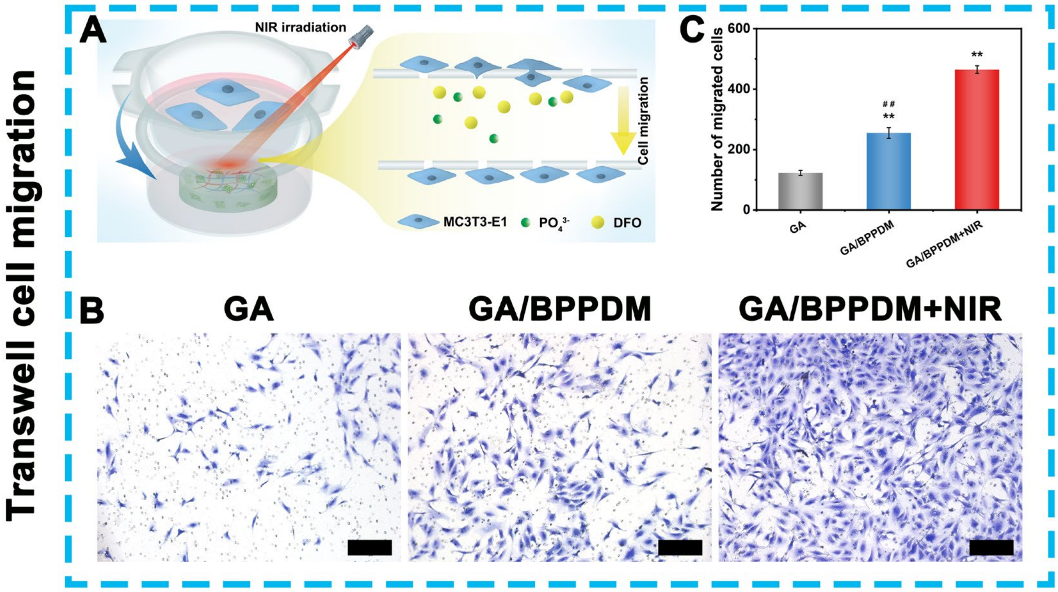

Fig. S16. (A) Schematic diagram of exploring the migration of MC3T3-E1 cells in a Transwell assay. **(B)** Crystal violet staining and **(C)** quantitative analysis of migrated MC3T3-E1 cells. Scale bar: 200 μm (B). Data are presented as the mean ± SD (n = 3). *P < 0.05 and **P < 0.01 indicate significant differences compared with the GA group. #P < 0.05 and # #P < 0.01 indicate significant differences compared with the GA/BPPDM+NIR group.

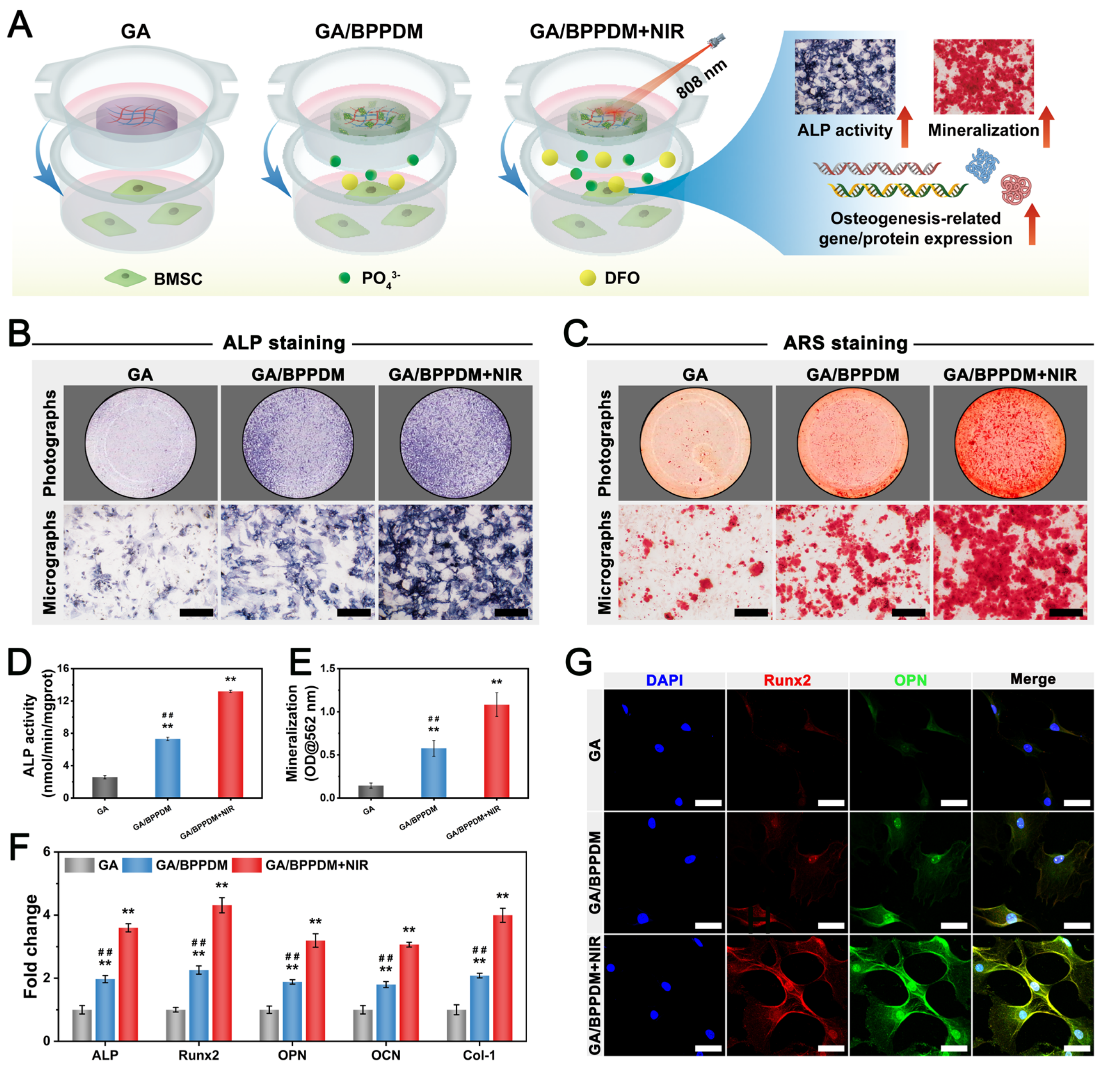

Fig. S17. (A) Schematic diagram of exploring the effect of GA/BPPDM hydrogels combined with NIR irradiation on the osteogenic differentiation of rat BMSCs. Macroscopic and microscopic images of **(B)** ALP staining and **(C)** ARS staining of BMSCs. Quantitative analysis of **(D)** ALP activity and **(E)** ARS staining. **(F)** Relative mRNA expression of osteogenesis-related genes in BMSCs, including ALP, Runx2, Col-1, OPN, and OCN. **(G)** Immunofluorescence staining of Runx2 and OPN in BMSCs. Scale bar: 200 μm (B, C), 40 μm (G). Data are presented as the mean ± SD (n = 3). *P < 0.05 and **P < 0.01 indicate significant differences compared with the GA group. $^{\#}$P < 0.05 and $^{\#\#}$P < 0.01 indicate significant differences compared with the GA/BPPDM+NIR group.

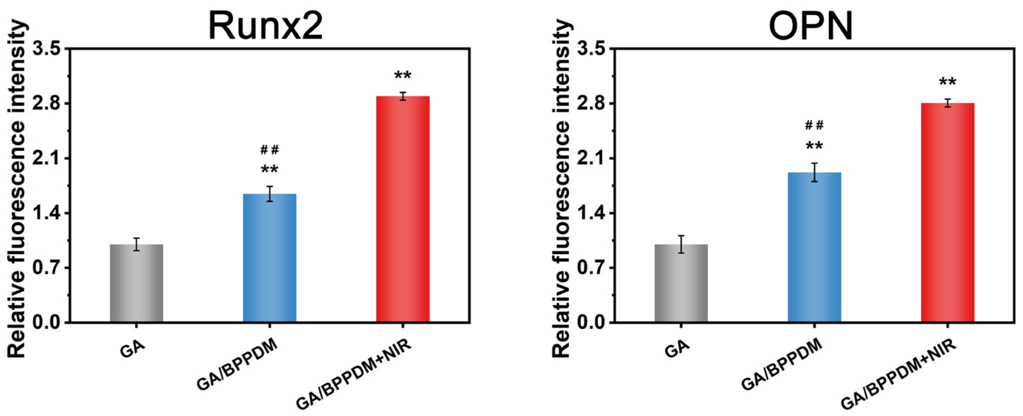

Fig. S18. Quantitative analysis of immunofluorescence staining of Runx2 and OPN in MC3T3-E1 cells. Data are presented as the mean ± SD (n = 3). *P < 0.05 and **P < 0.01 indicate significant differences compared with the GA group. $^{\#}$P < 0.05 and $^{\#\#}$P < 0.01 indicate significant differences compared with the GA/BPPDM+NIR group.

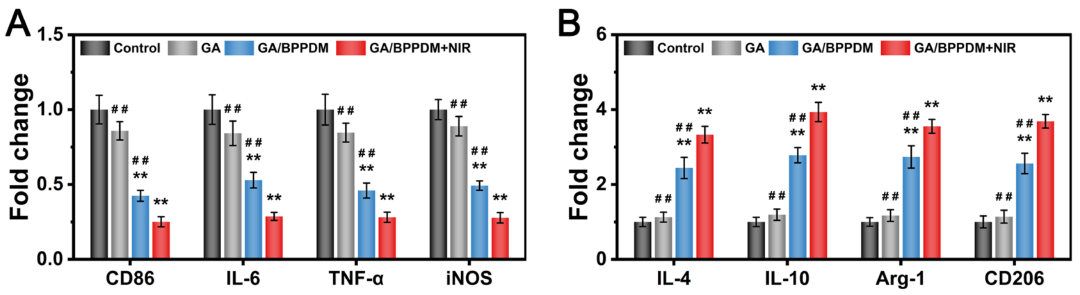

Fig. S19. Relative mRNA expression of **(A)** M1 (CD86, IL-6, TNF-α, and iNOS) and **(B)** M2 (IL-4, IL-10, Arg-1, and CD206) macrophage markers after implantation for 7 days. Data are presented as the mean ± SD (n = 3). *P < 0.05 and **P < 0.01 indicate significant differences compared with the control group. $^{\#}$P < 0.05 and $^{\#\#}$P < 0.01 indicate significant differences compared with the GA/BPPDM+NIR group.

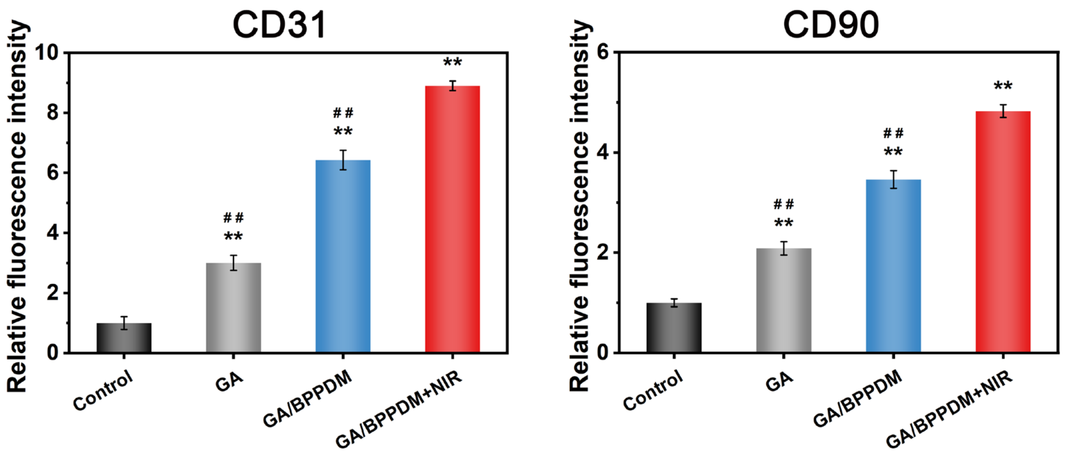

Fig. S20. Quantitative analysis of the fluorescence intensity of CD31 and CD90. Data are

presented as the mean ± SD (n = 3). *P < 0.05 and **P < 0.01 indicate significant differences compared with the control group. #P < 0.05 and ##P < 0.01 indicate significant differences compared with the GA/BPPDM+NIR group.

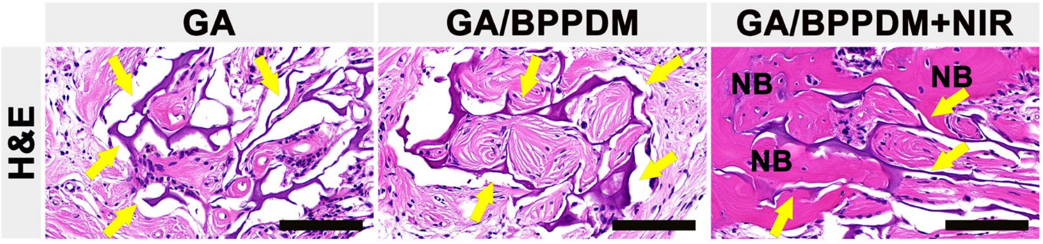

Fig. S21. H&E staining of regenerated bone around the hydrogel at 8 weeks after implantation. NB: newly formed bone tissue. Yellow arrows represent the residual hydrogel within the defect region. Scale bar: 100 μm.

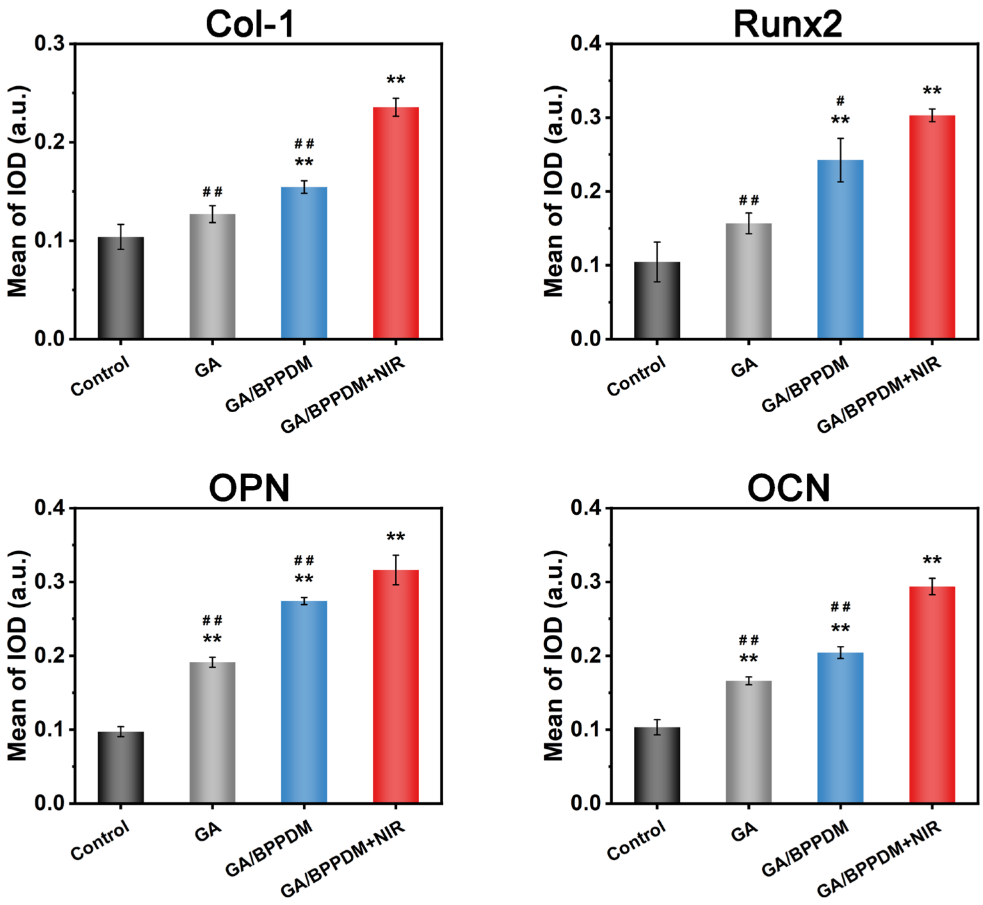

Fig. S22. Quantitative analysis of immunohistochemical staining of Col-1, Runx2, OPN, and OCN. Data are presented as the mean ± SD (n = 3). *P < 0.05 and **P < 0.01 indicate significant differences compared with the control group. #P < 0.05 and ##P < 0.01 indicate significant differences compared with the GA/BPPDM+NIR group.

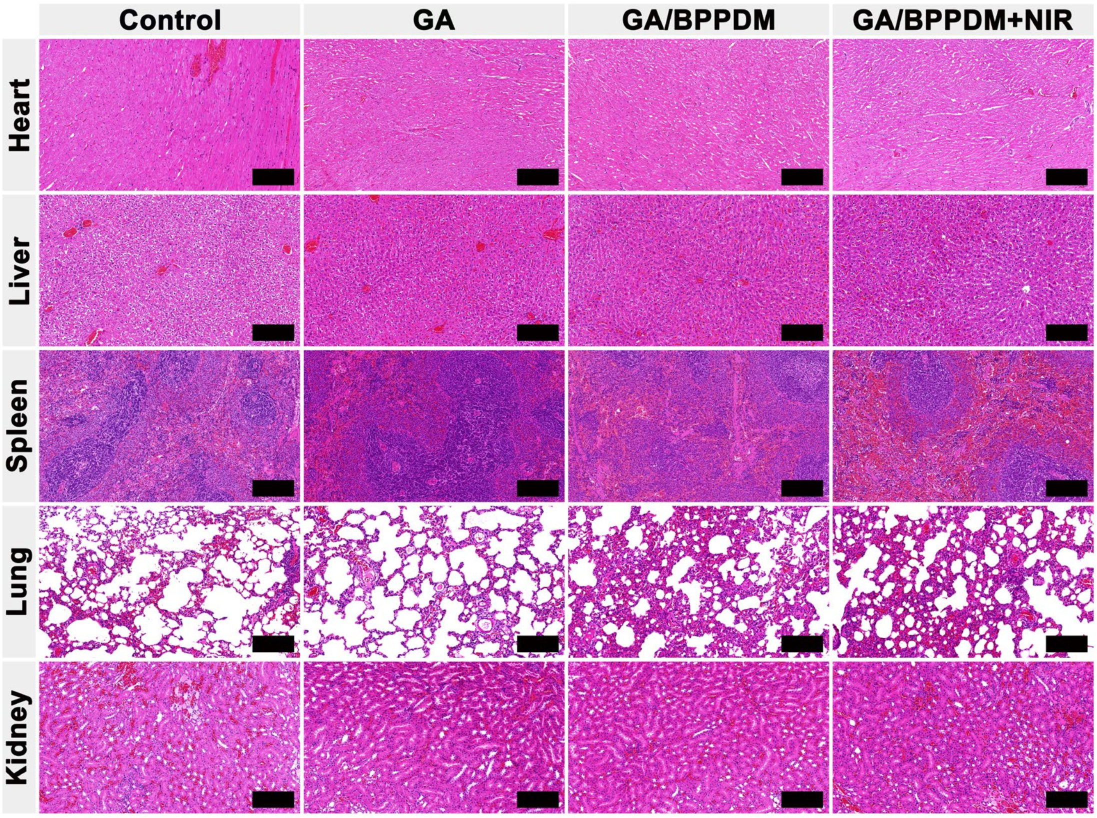

Fig. S23. H&E staining of major organs, including the heart, liver, spleen, lung, and kidney, of rats at 8 weeks after implantation. Scale bar: 200 μm.

Table S1. Composition for GA/BPPD hydrogels

Composition	DI H_2O	GelMA	Alg-MA	BPPD
GA	1 mL	70 mg	30 mg	0 mg
GA/BPPDL	1 mL	70 mg	30 mg	0.2 mg
GA/BPPDM	1 mL	70 mg	30 mg	0.5 mg
GA/BPPDH	1 mL	70 mg	30 mg	1.0 mg

Table S2. Primer sequences used in qRT-PCR analysis.

Genes	Primers (F, forward; R, reverse; 5’-3’)
Mouse-GADPH	F: TCAACGGCACAGTCAAGG<br>R: TTAGTGGGGTCTCGCTCC
Mouse-ALP	F: GAGCGTCATCCCAGTGGAG<br>R: TAGCGGTTACTGTAGACACCC
Mouse-Runx2	F: CATCCCAGTATGAGAGTAGGTGT

	R: GCTCAGATAGGAGGGGTAAGAC
Mouse-Col-1	F: CTGACTGGAAGAGCGGAGAG<br>R: CGGCTGAGTAGGGAACACAC
Mouse-OPN	F: TCTGAGGGACTAACTACGACCAT<br>R: TGGAAGAGTTTCTTGCTTAAAGTC
Mouse-OCN	F: TTCTGCTCACTCTGCTGACCC<br>R: CTGATAGCTCGTCACAAGCAGG
Mouse-TNF-α	F: CAGGCGGTGCCTATGTCTC<br>R: CGATCACCCCGAAGTTCAGTAG
Mouse-IL-6	F: GAGACCACTGGGGAGAATGC<br>R: TTGCCAGGTGGGTAAAGTGG
Mouse-iNOS	F: GAATCTTGGAGCGAGTTG<br>R: CCAGGAAGTAGGTGAGGG
Mouse-CD86	F: ATGGGCTCGTATGATTGT<br>R: TCTTAGGTTTCGGGTGAC
Mouse-IL-4	F: CATCCTGCTCTTCTTTCTC<br>R: TTCTCCTGTGACCTCGTT
Mouse-IL-10	F: TTTCAAACAAAGGACCAG<br>R: GGATCATTTCCGATAAGG
Mouse-Arg-1	F: AAGACAGCAGAGGAGGTG<br>R: AGTCAGTCCCTGGCTTA
Mouse-CD206	F: GCAAGTGATTTGGAGGCT<br>R: ATAGGAAACGGGAGAACC
Rat-GAPDH	F: CTCCCATTCTTCCACCTTTG<br>R: TGGTCCAGGGTTTCTTACT
Rat-CD86	F: CGAACACTATTTGGGCGCAG<br>R: CAAACTGGGGCTGCGAAAAA
Rat-IL-6	F: CCAGTTGCCTTCTTGGGACT<br>R: TCTGACAGTGCATCATCGCT
Rat-iNOS	F: CAGCTGGGCTGTACAAACCTT<br>R: CATTGGAAGTGAAGCGTTTCG
Rat-TNF-α	F: AGCACAGAAAGCATGATCCG<br>R: CACCCCGAAGTTCAGTAGACA
Rat-IL-4	F: ACCTTGCTGTCACCCTGTTC<br>R: TTGTGAGCGTGGACTCATTC
Rat-Arg-1	F: GGACATCGTGTACATCGGCT<br>R: TTTGCTGTGATGCCCCAGAT
Rat-IL-10	F: GTTCCCCTACTGTCATCCCC<br>R: AGGCAGACAAACAATACACCA
Rat-CD206	F: GGCTGATTACGAGCAGTGGA<br>R: CATCACTCCAGGTGAACCCC
Rat-Runx2	F: CCCAACTTCCTGTGCTCCGT<br>R: AAACTCTTGCCTCGTCCGCT
Rat-OPN	F: TCACTGCCAGCACACAAG<br>R: GCATCGGGATACTGTTCA
Rat-Col-1	F: CACTCAGCCCTCTGTGCCTC<br>R: AACCTTCGCTTCCATACTCG

Rat-OCN	F: TCTGCTCACTCTGCTGGC<br>R: TCCATTGTTGAGGTAGCG
Rat-ALP	F: AGAGAACTGCGGTTACTTAAACAT<br>R: TGTTCAGATCTGCCCCCATGAGAT
Human-GAPDH	F: CATCATCCCTGCCTCTACTGG<br>R: GTGGGTGTCGCTGTTGAAGTC
Human-VEGF	F: TATGCGGATCAAACCTCACCA<br>R: CACAGGGATTTTTCTTGTCTTGCT
Human-HIF-1α	F: ATCCATGTGACCATGAGGAAAT<br>R: CTCGGCTAGTTAGGGTACACTT
Human-bFGF	F: AAGAGCGACCCTCACATCAA<br>R: GCCAGGTAACGGTTAGCACA
Human-Ang-1	F: CAGGAGGATGGTGGTTTG<br>R: GCCCTTTGAAGTAGTGCC
Human-eNOS	F: ATGTTTGTCTGCGGCGATGT<br>R: GTGCGTATGCGGCTTGTC

References

1. Liu, X.; Li, L.; Gaihre, B.; Park, S.; Li, Y.; Terzic, A.; Elder, B. D.; Lu, L., *ACS nano* **2022,** *16* (2), 2741-2755.

2. Ma, Y.; Jiang, L.; Hu, J.; Zhu, E.; Zhang, N., *ACS applied materials & interfaces* **2022,** *14* (39), 44065-44083.

3. Zhang, J.; Tong, D.; Song, H.; Ruan, R.; Sun, Y.; Lin, Y.; Wang, J.; Hou, L.; Dai, J.; Ding, J.; Yang, H., *Advanced Materials* **2022,** *34* (36), 2202044.

4. Liu, H.; Chen, F.; Zhang, Y.; Wu, P.; Yang, Z.; Zhang, S.; Xiao, L.; Deng, Z.; Cai, L.; Wu, M., *Materials & Design* **2022,** *222*, 111070.

5. Wu, M.; Wang, Y.; Liu, H.; Chen, F.; Zhang, Y.; Wu, P.; Deng, Z.; Cai, L., *Materials & Design* **2023,** *227*, 111705.

6. Xu, Y.; Xu, C.; He, L.; Zhou, J.; Chen, T.; Ouyang, L.; Guo, X.; Qu, Y.; Luo, Z.; Duan, D., *Bioactive materials* **2022,** *16*, 271-284.

7. Wu, M.; Chen, F.; Liu, H.; Wu, P.; Yang, Z.; Zhang, Z.; Su, J.; Cai, L.; Zhang, Y., *Materials Today Bio* **2022,** *17*, 100458.

8. Zhao, Y.; Tian, C.; Liu, Y.; Liu, Z.; Li, J.; Wang, Z.; Han, X., *Biomaterials* **2023,** *295*, 122029.

9. Liu, H.; Shi, Y.; Zhu, Y.; Wu, P.; Deng, Z.; Dong, Q.; Wu, M.; Cai, L., *ACS applied materials & interfaces* **2023,** *15* (9), 12273-12293.

10. Luo, X.; Zhang, L.; Luo, Y.; Cai, Z.; Zeng, H.; Wang, T.; Liu, Z.; Chen, Y.; Sheng, X.; Mandlate, A. E. d. G.; Zhou, Z.; Chen, F.; Zheng, L., *Advanced functional materials n/a* (n/a),

2214036.
11. Li, X.; Xu, K.; He, Y.; Tao, B.; Li, K.; Lin, C.; Hu, J.; Wu, J.; Wu, Y.; Liu, S.; Peng, L.; Wang, H.; Cai, K., *Biomaterials* **2022,** *287*, 121683.
12. Xing, D.; Zuo, W.; Chen, J.; Ma, B.; Cheng, X.; Zhou, X.; Qian, Y., *ACS applied materials & interfaces* **2022,** *14* (33), 37380-37395.
13. Liu, H.; Shi, Y.; Zhu, Y.; Wu, P.; Deng, Z.; Dong, Q.; Wu, M.; Cai, L., *ACS applied materials & interfaces* **2023**.
